# In Situ Partial-Cyclized Polymerized Acrylonitrile-Coated NCM811 Cathode for High-Temperature ≥ 100 °C Stable Solid-State Lithium Metal Batteries

**DOI:** 10.1007/s40820-025-01683-7

**Published:** 2025-03-19

**Authors:** Jiayi Zheng, Haolong Jiang, Xieyu Xu, Jie Zhao, Xia Ma, Weiwei Sun, Shuangke Liu, Wei Xie, Yufang Chen, ShiZhao Xiong, Hui Wang, Kai Xie, Yu Han, Maoyi Yi, Chunman Zheng, Qingpeng Guo

**Affiliations:** 1 College of Aerospace Science and Engineering, National University of Defense Technology, Changsha, 410073 People’s Republic of China; 2 Changsha New Energy Innovation Institute, Changsha, 410083 People’s Republic of China; 3 State Key Laboratory for Mechanical Behavior of Materials, Xi’an Jiaotong University, Xi’an, 710049 People’s Republic of China; 4 Faculty of Materials Science and Engineering, Kunming University of Science and Technology, Kunming, 650093 People’s Republic of China

**Keywords:** Solid-state lithium metal battery, Ni-rich cathode, Interface engineering, In situ partial-cyclized PAN, High-temperature resistance

## Abstract

High-nickel ternary cathodes hold a great application prospect in solid-state lithium metal batteries to achieve high-energy density, but they still suffer from structural instability and detrimental side reactions with the solid-state electrolytes. To circumvent these issues, a continuous uniform layer polyacrylonitrile (PAN) was introduced on the surface of LiNi_0.8_Mn_0.1_Co_0.1_O_2_ via in situ polymerization of acrylonitrile (AN). Furthermore, the partial-cyclized treatment of PAN (cPAN) coating layer presents high ionic and electron conductivity, which can accelerate interfacial Li^+^ and electron diffusion simultaneously. And the thermodynamically stabilized cPAN coating layer cannot only effectively inhibit detrimental side reactions between cathode and solid-state electrolytes but also provide a homogeneous stress to simultaneously address the problems of bulk structural degradation, which contributes to the exceptional mechanical and electrochemical stabilities of the modified electrode. Besides, the coordination bond interaction between the cPAN and NCM811 can suppress the migration of Ni to elevate the stability of the crystal structure. Benefited from these, the In-cPAN-260@NCM811 shows excellent cycling performance with a retention of 86.8% after 300 cycles and superior rate capability. And endow the solid-state battery with thermal safety stability even at high-temperature extreme environment. This facile and scalable surface engineering represents significant progress in developing high-performance solid-state lithium metal batteries.

**Supplementary Information:**

The online version contains supplementary material available at 10.1007/s40820-025-01683-7.

## Highlights

- Uniform and stable interfacial layer with both ionic and electronic conduction on the surface of solid-state composite cathode by in situ polymerization cyclization treatment.
- Theoretical calculations demonstrate that cPAN can effectively inhibit transition metal dissolution and uneven cyclic stress distribution and improve the stability of the crystal structure.
- In-cPAN-260@NCM811 has excellent cycling performance with 86.8% retention after 300 cycles and thermally safe stability at high-temperature extremes.

**Supplementary Information:**

The online version contains supplementary material available at 10.1007/s40820-025-01683-7.

## Graphical abstract

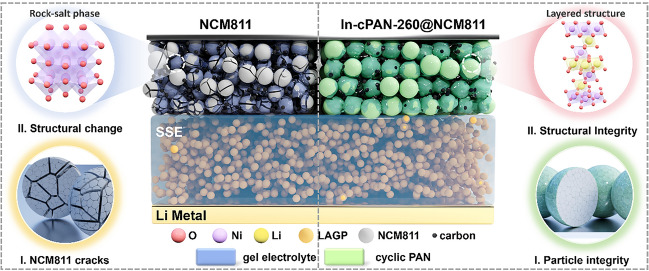

## Introduction

Lithium-ion batteries (LIBs) face the development requirements of high-energy density and high safety. Besides, LIBs are required to operate under extreme temperature in specialized fields such as underground exploration, aerospace, and weaponry. However, high-temperature working environments can exacerbate the risk of thermal runaway in batteries due to the flammability of traditional liquid electrolytes and exothermic decomposition of active materials [1–3]. Solid-state lithium batteries (SSLBs) considered to be the most prospective next-generation battery technology with high theoretical energy density and high safety even at high temperature [4–7]. And solid-state electrolytes (SEs) act as a critical component in SSLBs, which becomes a research hotspot [8, 9]. Among various SEs, poly(vinylidene fluoride-co-hexafluoropropylene) (PVDF-HFP) based electrolytes have been widely researched and demonstrated to be promising electrolyte system due to their high ionic conductivity and good electrochemical stability [10, 11]. However, the severe cathode interface issues between high-voltage cathode and PVDF-HFP based electrolytes still hinder the further development of the SSLBs. And high-temperature working environments can accelerate the deterioration of the cathode interface, resulting in the failure of SSLBs. Thus, the promise of SSLBs has stimulated extensive research for constructing excellent cathode interface with high-voltage cathode to meet the increasing demands of high-energy–density and high safety SSLBs [12].

Among various high-voltage cathode materials, the layered oxide cathodes, especially the Ni-rich NCM cathodes (e.g., LiNi_0.8_Mn_0.1_Co_0.1_O_2_, NCM811) are considered as the ideal material in SSLBs due to their low cost and high specific capacity [13, 14]. However, the high-nickel content and the further increase of charging voltage have caused many problems, such as poor thermal stability, structural degradation, and the detrimental side reactions with solid-state electrolytes [15–20]. To overcome these problems, introducing an inert modified layer on the surface of cathode particles is a highly effective strategy [21–25]. However, traditional coating methods are hard to achieve uniform and ultrathin coating layer on the particle surface. Additionally, most modified materials usually suffer from low electronic or ionic conductivity, resulting in limited improvements in the electrochemical performances [26]. Thus, a rational surface structure engineering that can resolve all of the aforementioned interfacial problems in SSLBs without sacrificing the transport of electrons and lithium ions during the process of charging-discharging is highly desirable.

**Fig. 1:**
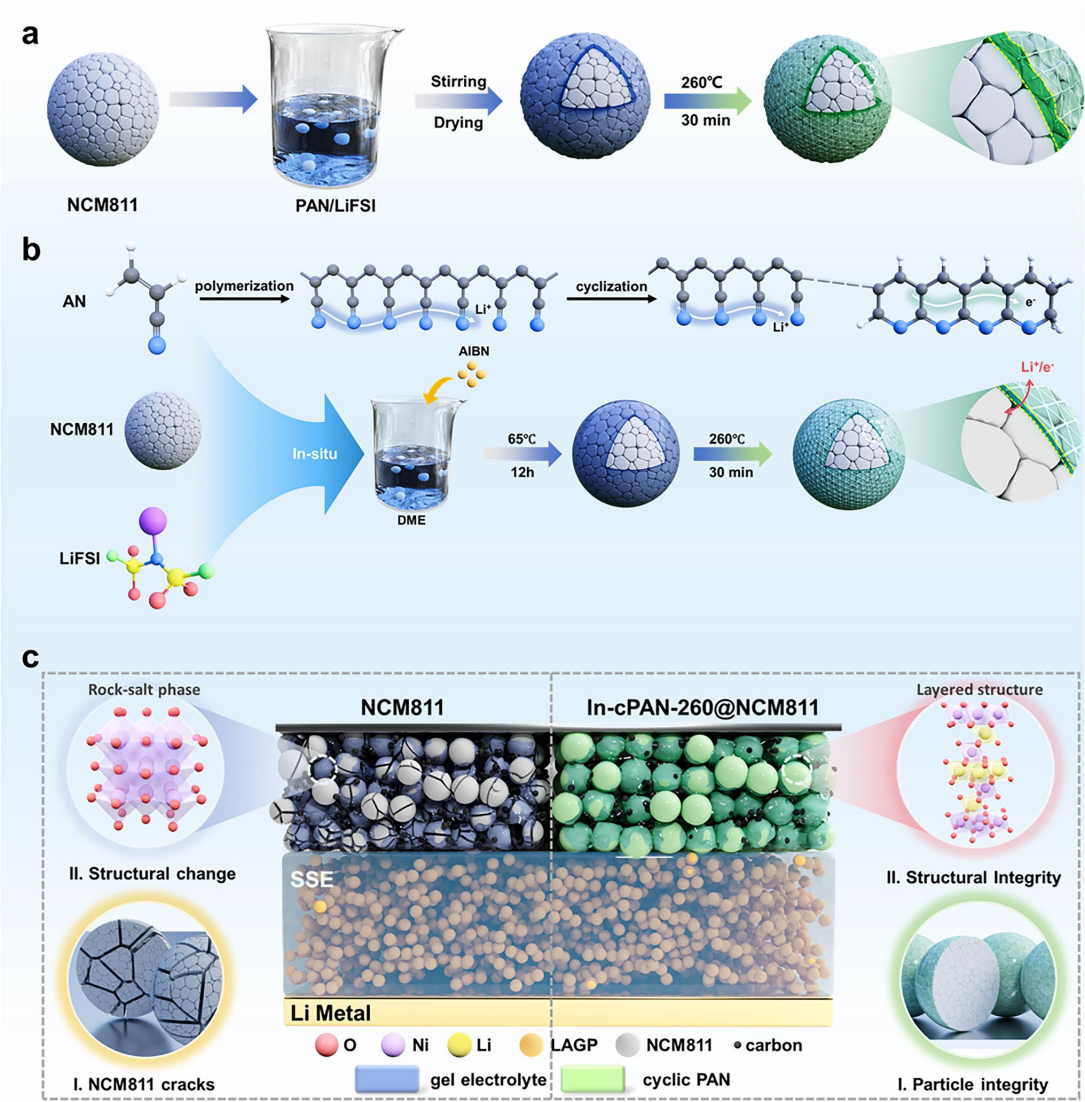
The schematic diagram of the preparation of In-cPAN@NCM811 cathode materials and solid-state lithium metal batteries. **a, b** Differences in the construction of cPAN coating layers on the NCM811 surface with generally wet coating and in situ polymerization. **c** Schematic illustration of the Li||NCM811and Li||In-cPAN@NCM811 solid-state batteries models at multiple scales

Herein, it is interesting to note that the partial-cyclized polyacrylonitrile (cPAN) exhibits excellent ionic and electronic conductivity characteristics, meanwhile, cPAN has high oxidation potential and dielectric constant, which can reduce occurrence of interface side reactions with cathode under high voltage [19]. With these in mind, we designed the strategy to utilize the bonding effect between the cyano group and transition metal cations in the high-nickel cathode, realizing a thinner, uniform, and controllable in situ polymerization cPAN coating layer on the NCM811 surface [27, 28], as shown in Fig. 1. Thus, the coating layers construct “high ionic/electronic” dual-conducting soft interfacial transmission path and endows stable and low interfacial resistance. More notably, the thermodynamically stabilized cPAN coating can not only effectively inhibit detrimental side reactions but also simultaneously address the problems of undesired phase transformations, intergranular and intragranular cracking. Benefitting from the above advantages, the optimized self-standing solid composite cathode exhibit remarkably improved electrochemical performance, excellent electrochemical stability, even under exertive operational conditions at elevated temperature and nail penetration test. Therefore, this facile and scalable surface engineering provides another possibility for the high-energy density and high security solid-state lithium metal batteries.

## Experimental Section

### Preparation of Materials and Solid-State Composite Cathode

A small amount of PAN samples was taken into crucibles, heated to 240, 260, and 280 °C at the heating rate of 0.5 °C min^−1^ in a muffle furnace in air atmosphere, and fully calcined for 0.5 h. The samples treated at different temperatures were named cPAN-240, cPAN-260, and cPAN-280, respectively.

Using azobisisobutyronitrile (AIBN) as initiator, acrylonitrile (AN) can be polymerized with comonomer methyl acrylate (MA) in dimethyl ether (DME) at 65 °C for 12 h to form polyacrylonitrile (PAN). The mass fraction of polymer monomer AN in solution was 30%, the mass ratio of AN to MA was 9:1, and the amount of initiator AIBN was 0.6%. In order to realize the in situ polymerization of PAN on the cathode surface and form an ion conducting phase, add NCM811 particles and lithium bis(fluorosulfonyl)imide (LiFSI) into the solvent. After polymerization, the solvent was directly evaporated and dried at 100 °C to obtain NCM811 material coated by in situ polymerization, in which the yield of PAN is about 33%. In order to obtain PAN coated NCM811 cathode materials with different cyclization structures, the samples were calcined at 260 °C for 0.5 h, and the heating rate is 0.5 °C min^−1^. The cyclized cathode materials are named as In-cPAN-260@NCM811.

An appropriate amount of PVDF was dissolved in N-methylpyrrolidone (NMP) at 60 °C with magnetic stirring for 30 min to obtain a 5.9% clarified solution of the polymer. Subsequently, lithium salt and ionic liquid were added to the polymer clarified solution under inert atmosphere conditions according to the mass ratio of = 5:5:7, and the solution was stirred continuously for 3 h to obtain a homogeneous mixed solution. The above prepared composite electrolyte slurry was mixed with the active substance In-cPAN-260@NCM811, conductive agent (conductive carbon KS-6 and superconducting carbon SP) and NMP, and ball milled for 30min to fully mix the components to obtain the cathode slurry. After uniform coating and drying at 110 °C under vacuum drying condition for 24 h, the In-cPAN-260@NCM811 composite cathode with 80% active substance content was obtained, in which the active substance surface loading was 2.44 mg cm^−2^.

### Preparation of Solid Composite Polymer Electrolyte

Poly(vinylidene fluoride-co-hexafluoropropylene) (PVDF-HFP) was dissolved in butanone solvent. In the solvent of butanone, the solution was heated and stirred at 60 °C for 30 min to obtain a clear solution with a concentration of 5%. Add LAGP electrolyte powder, lithium salt LiTFSI, ionic liquid EMITFSI into the glove box (m_PVDF-HFP_: m_LAGP_: m_LiTFSI_: m_EMITFSI_ = 5: 5: 5: 7), the solution is sealed and stirred for 3 h to obtain a uniform electrolyte slurry. The slurry was then poured into a stainless-steel mold, scraped evenly with a glass rod and transferred to a vacuum drying oven, and finally dried in a vacuum drying oven at 80 °C for 12 h to obtain a homogeneous electrolyte membrane. The electrolyte membrane was cut into disks with a diameter of 19 mm and stored in a glove box for spare use, and the thickness of the electrolyte membrane was about 50 μm.

### Materials Characterizations

The phase and crystal structure of the material were characterized by XRD on SIEMENS D-500 X-ray diffractometer with Cu Kα radiation with a scanning rate of 10°·min^–1^ and within a 2θ range between 10° and 80°. Scanning electron microscopy (SEM, HITACHI S-4800) and transmission electron microscopy (TEM, FEI Tecnai 2100) were used to characterize the structure and morphology of the NCM811 cathode before and after capping. The cyclization degree of PAN at various temperatures was characterized by FTIR on Thermo Fisher Scientific Nicolet iN10 with scanning 32 times between 400 and 4000 cm^−1^. The calculation formula is as follows:

1 $${I}_{C}=\frac{f\times ABS(1610+1585)}{ABS\left(2240\right)+f\times ABS(1610+1585)}$$

where *f* represents the ratio of cyano to cyclized group absorption constant, which is 0.29, ABS (1610 + 1585) represent the absorbance of cyclized structure C = N, C = C and N–H, and ABS (2240) represents the absorbance of C≡N.

The roughness of the the NCM811 cathode before and after coating was measured by atomic force microscopy (AFM, Bruker Dimension Icon). X-ray photoelectron spectroscopy (XPS, Thermo Scientific K-alpha) was used to characterize the changes in the chemical environment on the surface of the cathode before and after coating and before and after cycling, and the results were analyzed using Avantage software. Time-of-flight secondary ion mass spectrometry (TOF–SIMS, ION-TOF TOF.SIMS5) was used to analyze the chemical composition and depth distribution of the cathode after cycling. Accelerating rate calorimeter (ARC) adopts a heating wait search (H-W-S) mode to analyze the safety of solid-state and liquid batteries.

### Electrochemical Measurements

The battery performance was evaluated by performing constant current charge/discharge cycles on a LAND CT2001A test system for a 2032 button cell. The solid-state battery was assembled with In-cPAN-260@NCM811 as the cathode, lithium metal as the anode, and the prepared LAGP solid-state composite electrolyte as the solid-state electrolyte. Constant current charge/discharge cycle tests were performed in the voltage range of 3.3 ~ 4.3 V at 60 °C. Electrochemical impedance spectroscopy (EIS) tests were performed at Princetion Versa STAT electrochemical workstation with an applied voltage vibration amplitude of 10 mV and a test frequency range of 10^5^–0.01 Hz.

### DFT Calculation

The computations were performed using the first-principles calculation implementation of CASTEP [29]. The generalized gradient approximation (GGA) [30] with the PerdewBurke-Ernzerhof (PBE) formula [31] was used for the exchange-correlation potential in combination with the DFT-D correction. Spin-polarized calculations were employed. The Broyden-Fletcher-Goldfarb-Shanno (BFGS) method was used to search for the ground state of the supercells, and the convergence tolerance was set to the energy change below 10^−5^ eV per atom, force less than 0.02 eV Å^−1^, stress less than 0.05 GPa, and displacement change less than 0.001 Å. The cutoff energy of the atomic wave functions was set to 520 eV.

### Multiphysics Simulations

The stress generated by the delithiation/lithiation of AM inside the composite NCM cathode can be described using the quasi-static equilibrium with the neglection of mechanical body forces [32–34]:

2 $$ \nabla  \cdot \sigma  = 0 $$

3 $$ \varepsilon  = \left[ {\frac{1}{2}\left( {\nabla u} \right) + \left( {\nabla u} \right)^{T} } \right] $$

Where σ is Cauchy stress tensor calculated by Hook’s law, ε is the elastic strain, and u is the displacement. Thus, the strain inside AM particles εAM can be yielded as the proportion of the lithiation fraction based on the isotropic assumption to calculate the deformation of secondary particles for simplicity:

4 $$ \varepsilon _{{AM}}  = \frac{1}{3}tr\left( \varsigma  \right)c_{{\max }}^{{AM}} \left( {1 - x} \right) $$

Furthermore, Xu-Needleman cohesive zone model was conducted to investigate the mechanical breakdown of secondary AM particles with different primary particles. First, the virtual work can be given including the terms of specimen volume (*V*), internal surface (*S*_*in*_), and external surface (*S*_*ex*_):

5 $$ \int\limits_{V} {\mathbf{s}:\delta \mathbf{F}dv}  - \int\limits_{{\text{s} _{{in}} }} {\mathbf{T} \cdot \delta \Delta ds}  = \int\limits_{{\text{s} _{{ex}} }} {\mathbf{T} \cdot \delta \mathbf{u}ds}  + \int\limits_{{\text{s} _{{ex}} }} {\rho \frac{{\partial ^{2} u}}{{\partial t^{2} }}\delta \mathbf{u}ds}  $$

With nonsymmetric nominal stress tensor, *s* = *F*^−1^*det*(***F***)**σ**, displacement gradient, ***F***, the displacement jumps across the cohesive surface, Δ = (Δ_*n*_***n*** + Δ_*t*_***t***) and density, *ρ*. ***T*** is the traction vector on the surface related to Cauchy stress, described as ***T = σ⋅n.*** Combined with exponential law from Beltz and Rice, normal traction (*T*_*n*_) and tangential traction (*T*_*t*_) can be yielded as [35, 36]:

6 $$ T_{n}  = \sigma _{{\max ,0}} e\exp \left( { - \frac{{\Delta u_{n} }}{{\delta _{0} }}} \right)\left\{ { - \frac{{\Delta u_{n} }}{{\delta _{0} }}\exp \left( {\frac{{\Delta u_{t}^{2} }}{{\delta _{0}^{2} }}} \right) + \left( {1 - q} \right)\frac{{\Delta u_{n} }}{{\delta _{0} }}\left[ {1 - \exp \left( { - \frac{{\Delta u_{t}^{2} }}{{\delta _{0}^{2} }}} \right)} \right]} \right\} $$

7 $$ T_{n}  = 2\sigma _{{\max ,0}} eq\frac{{\Delta u_{t} }}{{\delta _{0} }}\left( {1 + \frac{{\Delta u_{t} }}{{\delta _{0} }}} \right)\exp \left( { - \frac{{\Delta u_{t} }}{{\delta _{0} }} - \frac{{\Delta u_{t}^{2} }}{{\delta _{0}^{2} }}} \right) $$

With the initial cohesive strength for the maximum normal traction of *σ*_*max*,0_ and cohesive length of *δ*_*o*_.

## Results and Discussion

### Structure and Characterization

**Fig. 2:**
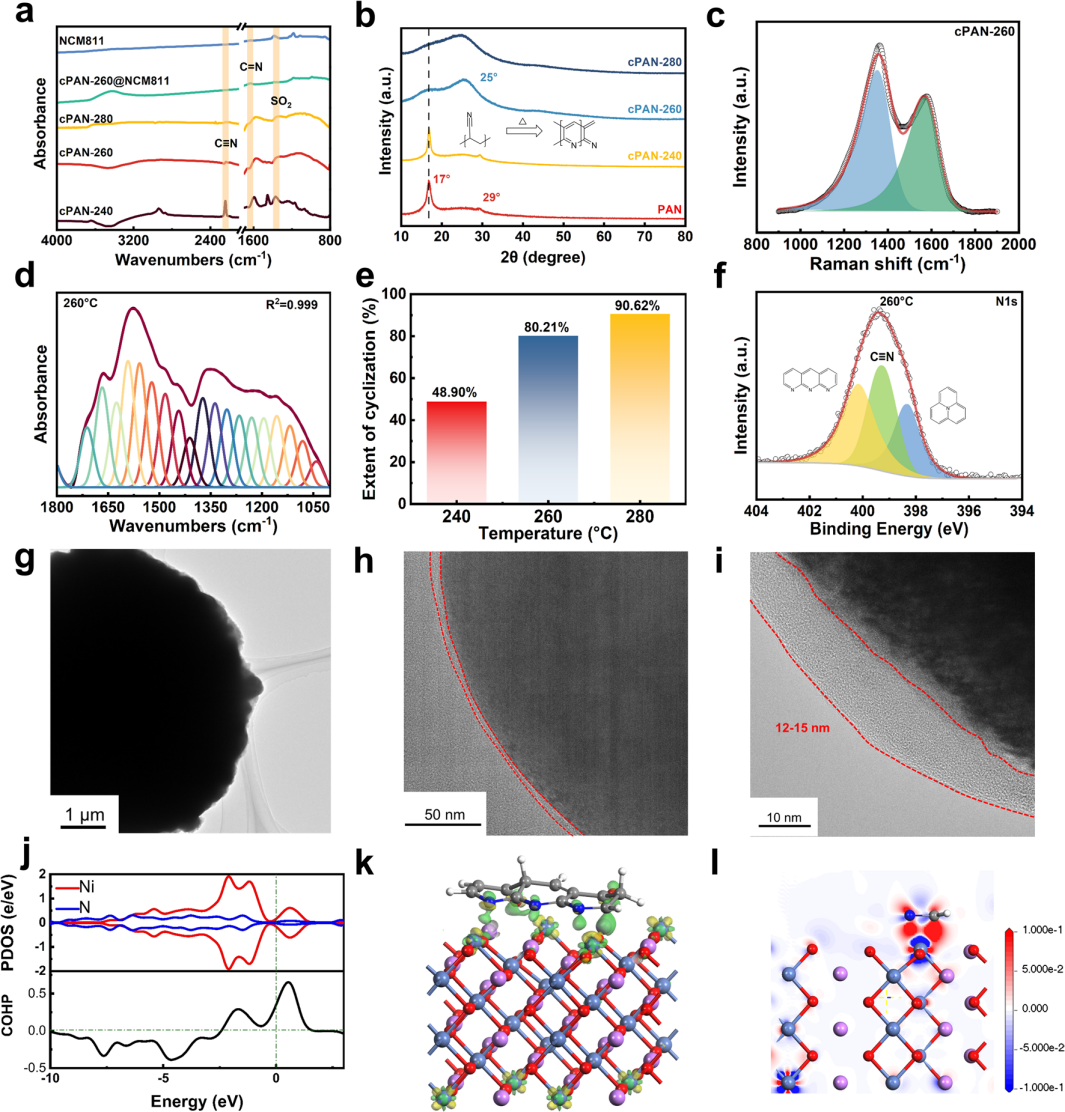
Coating of in situ cPAN on surface of NCM811 cathode. **a** FTIR pattern of cPAN formed at different temperature. **b** XRD pattern of cPAN formed at different temperature. **c** Raman spectra of cPAN-260. **d** Split-peak fitting curve of 1800–1000 cm^−1^ for the infrared spectrum of cPAN260. **e** Cyclization rate of PAN at different temperatures. **f** XPS spectra of cPAN-260. **g-i** TEM images of In-cPAN-260@NCM811. **j** Calculated PDOS and COHP of N-Ni type binding structures. **k** Side view of the In-cPAN-260@NCM811 surface with N atoms binding to Ni atoms. **l** Two-dimensional charge density difference of the In-cPAN-260@NCM811 surface with N atoms binding to Ni atoms

The reaction process of the in situ polymerization PAN had been systemically studied, as shown in Fig. 2. The infrared spectrum such as the characteristic peaks and shoulder peaks at 1385 and 1363 cm^−1^ are derived from the stretching vibration of SO_2_ (Fig. 2a), which consists with LiFSI, while the characteristic peak at 2245 cm^−1^ belongs to the stretching vibration peak of cyanide group in in situ PAN. The diffraction peaks of in situ PAN in XRD can match well with that of commercial PAN (Fig. S1), indicating the successfully synthesized of PAN. Simultaneously, there exist large numbers of amorphous regions in both types of polyacrylonitrile, and the disordered phase is distributed throughout the entire structure in non-discrete manner.

As shown in Fig. S2, the ionic conductivity of the in situ PAN coating layer can reach 1.22 × 10^–4^ S cm^−1^ at 25 °C. Meanwhile, in order to endow PAN with high electronic conductivity, the high-temperature cyclization reaction process of PAN (cPAN) had been studied. Firstly, with the increase of heat treatment temperature, the color of PAN powder gradually deepened (Fig. S3), indicating that PAN was cross-linked and cyclized to form heterocyclic molecules during the pre-oxidation process of heat treatment in air atmosphere. Specifically, XRD was used to analyze the change of PAN structure during this process, as shown in Fig. 2b, the samples of cPAN-240 still exhibits a strong diffraction peak at approximately 17° and a weak diffraction peak near 29°. As the temperature increases, the peak of cPAN-260 at about 17° disappears and transforms into a broad peak, exhibiting a noticeable broad peak near 25°, which is consistent with the (002) crystal plane of quasi-graphite structure. This means that the cyclization of the cyano group results in a graphitized structure during heat treatment, and there is a large layer spacing between adjacent stepped structures [37]. Further, the changes of PAN molecular structure and composition with different heat treatment temperature were analyzed by Raman spectroscopy. In Figs. 2c and S4, the Raman spectra showed typical D-peaks (1362 cm^−1^) and G-peaks (1570 cm^−1^) after heat treatment, indicating the coexistence of that the *sp*^3^ hybrid C–C (or C-N) single bond and *sp*^2^ hybrid C = C (or C = N) double bond in the heat treatment PAN materials. This confirms the formation of a delocalized conjugated heterocyclic structure in the cPAN after heat treatment. The *I*_D_/*I*_G_ values of the cPAN materials after heat treatment at 240, 260, and 280 °C were 2.06, 2.27, and 2.49, respectively. It is evident that as the heat temperature increases, the component of C = C (or C = N) in cPAN increased, as well as the degree of graphitization. In order to effectively quantify the degree of PAN cyclization, the infrared absorption peaks of C = N, C = C, and C-H groups are separated from the complex overlapping peaks in the range of 1800–1000 and 2270–2170 cm^−1^ by peak splitting fitting (Fig. 2d). Hereafter, the degree of PAN cyclization can be calculated based on Eq. (1) in support information, according to the infrared spectrum data. As shown in Fig. 2e, the cyclization degree of PAN is from 48.9% (240 °C) to 80.21% (260 °C) and then to 90.62% (280 °C). XPS spectra was used to further analyze the cyclization structure and degree of cPAN at different heat treatment temperatures. Figures 2f and S5 are the N 1*s* spectra of cPAN at different heat treatment temperatures. For cPAN-240, the characteristic peak at 398.8 eV is attributed to pyridine nitrogen, while the characteristic peak with binding energy of 400 eV may be graphite nitrogen or pyrrole nitrogen [38], both of which are generated by the cyclization and cross-linking of PAN molecules. The characteristic peak of 399.7 eV corresponds to the carbon nitrogen triple bond of the cyano group in PAN. Based on the peak areas of the three characteristic peaks, it is evident that cPAN-240 still contains a significant number of cyano groups and a low degree of cyclization. With an increase in heat treatment temperature, the cyano characteristic peak area in the N1s spectrum of cPAN-260 decreased significantly and changed into pyridine nitrogen and graphite nitrogen.

After clarifying the structural characteristics of cyclized PAN at different cyclization temperatures, the microstructure and surface characterization of coated NCM811 was further investigated. The XRD spectra of the pristine NCM811 and the in situ PAN@NCM811 are shown in Fig. S6; there are no impurity peaks in the samples before and after coating, indicating that the coating process has no effect on the crystal structure of NCM811. From the TEM images in Fig. S7, the thickness of NCM811 particles coated by the PAN with generally wet coating (PAN@NCM811) is about 20.4 nm, and the edge of the PAN@NCM811 particle is uneven. Comparatively, a uniform coating layer with an average thickness of about 12–15 nm on the surface of In-cPAN-260@NCM811 particles can be observed in Fig. 2g–i. The edges of the particles become blurred and surface roughness decreases distinctly after coating as shown in Fig. S7, which ranging from 24.8 nm of pristine NCM811 to 15.6 nm of In-cPAN-260@NCM811. Figure S8 shows the FTIR spectra of NCM811 and In-cPAN-260@NCM811, the characteristic peak of C = N at 1630 cm^−1^ and SO_2_ at 1385 cm^−1^ can be observed on the infrared spectrum of in situ cPAN@NCM811, which belong to cyclized polyacrylonitrile and lithium salt LiTFSI [39]. Meanwhile, the Raman spectra of the In-cPAN-260@NCM811 in Fig. S9 shows the expected D and G bands as cPAN-260, which confirms the presence of the in situ cPAN coating layer. At the same time, the four-probe test of the cathode showed that the electronic conductivity of the In-cPAN-260@NCM811 was 0.14 S cm^−1^, which is significantly higher than that of the uncoated cathode (0.0052 S cm^−1^). This is also consistent with the change of graphitization after cyclization treatment, thus, π electrons in PAN polymer matrix can be evenly distributed. Herein, considering the conduction of ions and electrons, the product with a heat treatment temperature of 260 °C may be in an ideal state of cyclization.

Furthermore, the interacting mechanism between NCM811 and cPAN was revealed via first-principles calculation. The cyano group, as an electron-rich group, forms a coordination interaction with transition metal ions. The partial density of states (PDOS) of Ni and N are shown in Fig. 2j; peak overlap can be observed in a relatively large energy range near the Fermi level, indicating the electron interaction between Ni and N. Furthermore, the integral of the Crystal Orbital Hamilton Population (iCOHP) can approximately analyze the strength of the Ni–N bond, which can support that the Ni–N bond has a certain bonding strength [40]. For exploring bonding types of Ni–N, charge density difference was carried out. As shown in Fig. 2k, green area represents electron density enrichment and yellow area represents electron density dissipation in the three-dimensional charge density difference. In the Ni–N bond region, the green area is more concentrated near N, and the yellow area is more inclined to Ni, showing the characteristics of coordination bonds. Besides, the two-dimensional charge density difference (Fig. 2l) reveals that the electron-depleted region (blue) is predominantly around Ni, while the electron-rich region (red) is clearly skewed toward N. Therefore, the uniform and ultrathin cPAN coating layer on the surface of NCM811 can be attributed to the formation of coordination bond between Ni and N.

### Electrochemical Performance

**Fig. 3:**
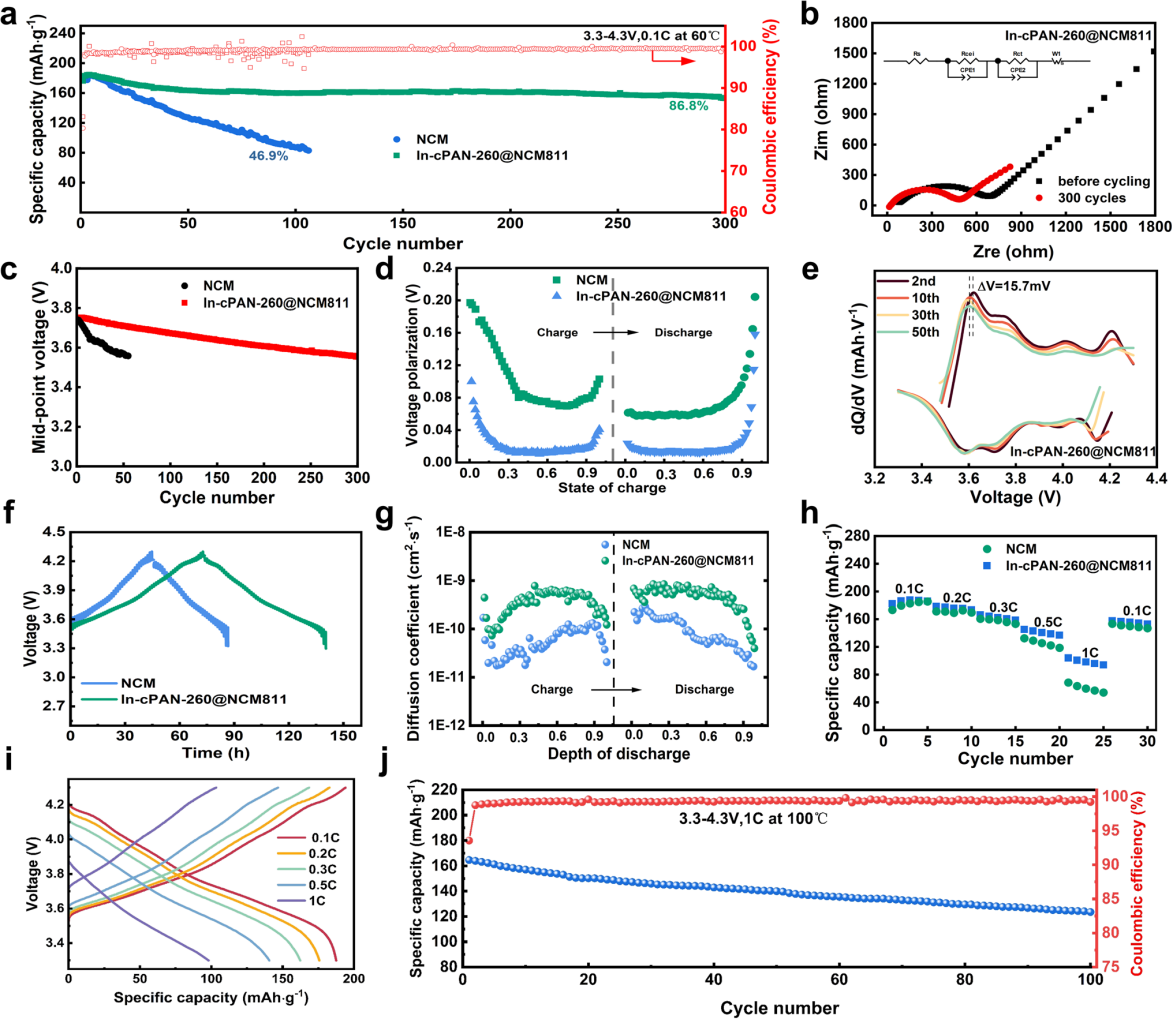
Electrochemical performance of solid-state battery with In-cPAN-260 cathode. **a** Cycle performance of In-cPAN-260@NCM811 at 60 °C. **b** Impedance spectra of In-cPAN-260@NCM811/SCPE/Li before and after cycling. **c** Discharge mid-point voltage curve. **d** Charging and discharging relaxation overpotential. **e** d*Q*/d*V* curves of In-cPAN-260@NCM811. **f** GITT curves of In-cPAN-260@NCM811 and NCM811. **g** Li^+^ diffusion coefficient of In-cPAN-260@NCM811 and NCM811. **h** Rate performance of In-cPAN-260@NCM811 and NCM811. **i** Charge and discharge curves of In-cPAN-260@NCM811 at different current density. **j** Cycling performance of In-cPAN-260@NCM811 at 100 °C

Herein, the electrochemical performances of the solid-state lithium metal batteries (SSLMBs) were recorded to verify the effect of the coating strategy. The basic electrochemical performances of PVDF-HFP-based solid composite polymer electrolyte (SCPE) are shown in Fig. S10 and S11. The used SCPE possesses high ionic conductivity of 0.44 mS cm^−1^ at room temperature, 0.78 mS cm^−1^ at 60 °C and presents above 4.5 V oxidation potential. At the current density of 0.05 mA cm^–2^ and the capacity of 0.05 mAh cm^–2^, the lithium symmetric cells with this electrolyte deliver a cycling life of 3000 h. The NCM811/SCPE/Li and In-cPAN-260@NCM811/SCPE/Li batteries were tested at high temperature 60 °C under a voltage range of 3.3–4.3 V. In-cPAN-260@NCM811 shows a superior cycling performance than NCM811 (Fig. 3a) at 0.1C. In addition to the significant capacity retention improvement, as shown in the charge–discharge voltage profiles during cycling (Fig. S12a, b), the voltage decay of In-cPAN-260@NCM811 is also markedly mitigated, indicating the suppression of the undesired structural transformation and side reaction in the interface. Furthermore, as shown in Fig. 3c, the initial median discharge voltage of In-cPAN-260@NCM811 solid-state battery is 3.75 V, which is slightly higher than that 3.74 V of NCM811. After 300 cycles, the median discharge voltage of the battery is 3.55 V with the voltage drop rate is 0.67 mV per cycle, which is significantly slower than that of NCM811 battery of 3.4 mV per cycle. In order to analyze the influence of the ion–electron mixed conduction interface constructed by cyclized PAN on the kinetics of Li^+^ migration during the long cycle, the electrochemical impedance spectra of In-cPAN-260@NCM811 before and after cycles at 4.3 V cutoff voltage were tested. As shown in Fig. 3b, the EIS can be specifically disassembled into four main components: the intrinsic impedance of the battery (*R*_s_), the resistance of Li^+^ migration through the interfacial membrane (*R*_CEI_), the charge transfer resistance between the electrode/electrolyte interface (*R*_ct_), and the diffusive migration impedance of Li^+^ in the active material (*Z*_w_). Notable, R_ct_ of In-cPAN-260@NCM811 decreases from 593.8 to 350.9 Ω after 300 cycles, which can be related to a compact CEI layer formed at the cathode–electrolyte interface during cycling. And the introduced interfacial layer can prevent the impact of insulating decomposition products on ion transport.

Based on these results, in order to verify the structural reversibility of In-cPAN-260@NCM811 during the electrochemical reactions, we further evaluated their differential capacity *versus* voltage (d*Q*/d*V*) profiles corresponding to selected charge/discharge cycles. During the process of charging, the three peaks of approximately 3.6, 4, and 4.2 V represent three pairs of phase transitions from hexagonal phase H1 to monoclinic phase M, monoclinic phase M to hexagonal phase H2, and hexagonal phase H2 to hexagonal phase H3, respectively. Compared with the pristine NCM811 (Fig. S12d), the capacity differential curves of the In-cPAN-260@NCM811 (Fig. 3e) show a good overlap in the first 50 cycles, indicating that the electrode cycle has high reversibility. During the second to 50th cycles, the H1-M oxidation peak of the In-cPAN-260@NCM811 composite cathode shifted only 15.7 mV to the left. Since the reversible capacity decay of high-nickel materials is closely related to the microcracks and structural degradation caused by the H2-H3 phase transition [41], the cPAN coating layer effectively protects the high-nickel cathode particles during the H2-H3 phase transition through the reduction of the side reaction and the stabilization of the rigid body-type network structure (inter-polymer cross-linking), which enhances the reversibility of the phase transition to achieve high capacity retention during the long-term cycling process. Simultaneously, the ion diffusion coefficient D_Li_^+^ of the different cathodes was measured by GITT, and the results are shown in Fig. 3(f, g). Compared to NCM, In-cPAN-260@NCM811 has a higher D_Li_^+^ and lower relaxation overpotential in the process of charge and discharge (Fig. 3d). This result indicates that the cyclized PAN-coated cathode surface has a faster charge transfer rate, which can be attributed to the high ion and electron conductivity of cPAN [42]. Meanwhile, In-cPAN-260@NCM811 presents superior rater performance and lower polarization voltage compared with NCM811 based on Figs. 3(h, i) and S12c, demonstrating that the In-cPAN-260 coating layer synergistically facilitates the rapid transport of Li ions and electron through the interface. Furthermore, it is worth noting that the In-cPAN-260@NCM811/SCPE/Li batteries present excellent cycling performance at 1C even if the operating temperature reaches up to 100 °C (Fig. 3j). To sum up, the In-cPAN-260 layer can enhance ion and electron transport inside the composite cathode and ensure the cycling stability of cathode even operated in high temperature.

In order to further evaluate the effect of In-cPAN-260 coating layer on the surface of NCM811, the in situ electrochemical impedance spectra was carried out to analyze the interface state during charge and discharge. As shown in Fig. S13, the pristine NCM811 shows obvious polarization during the first charging process, which was due to poor internal and interface contact affecting lithium-ion transport. During the first cycling process, the R_ct_ of NCM811 decreases firstly and gradually increases when the voltage is higher than 4.0 V, which may be affected by the change of Li^+^ concentration on the surface of NCM811 particles and the side reactions at the interface. Comparatively, In-cPAN-260@NCM811 cathodes show lower polarization at the first charging process, indicating that the introduction of coating layer by in situ polymerization has a significant effect on improving the lithium-ion migration and interface contact. And the *R*_CEI_ values are basically consistent during the two cycles, indicating that a stable CEI layer can be formed at the positive electrode interface in the early stages of the cycle. The above results indicate that in situ polymerization partial cyclization coating with high ion and electronic conductivity can form a stable interface layer during the cycling process, suppress the occurrence of side reactions, and improve the cycling stability of solid-state batteries.

### Electrochemical Mechanism of Coated Cathode

**Fig. 4:**
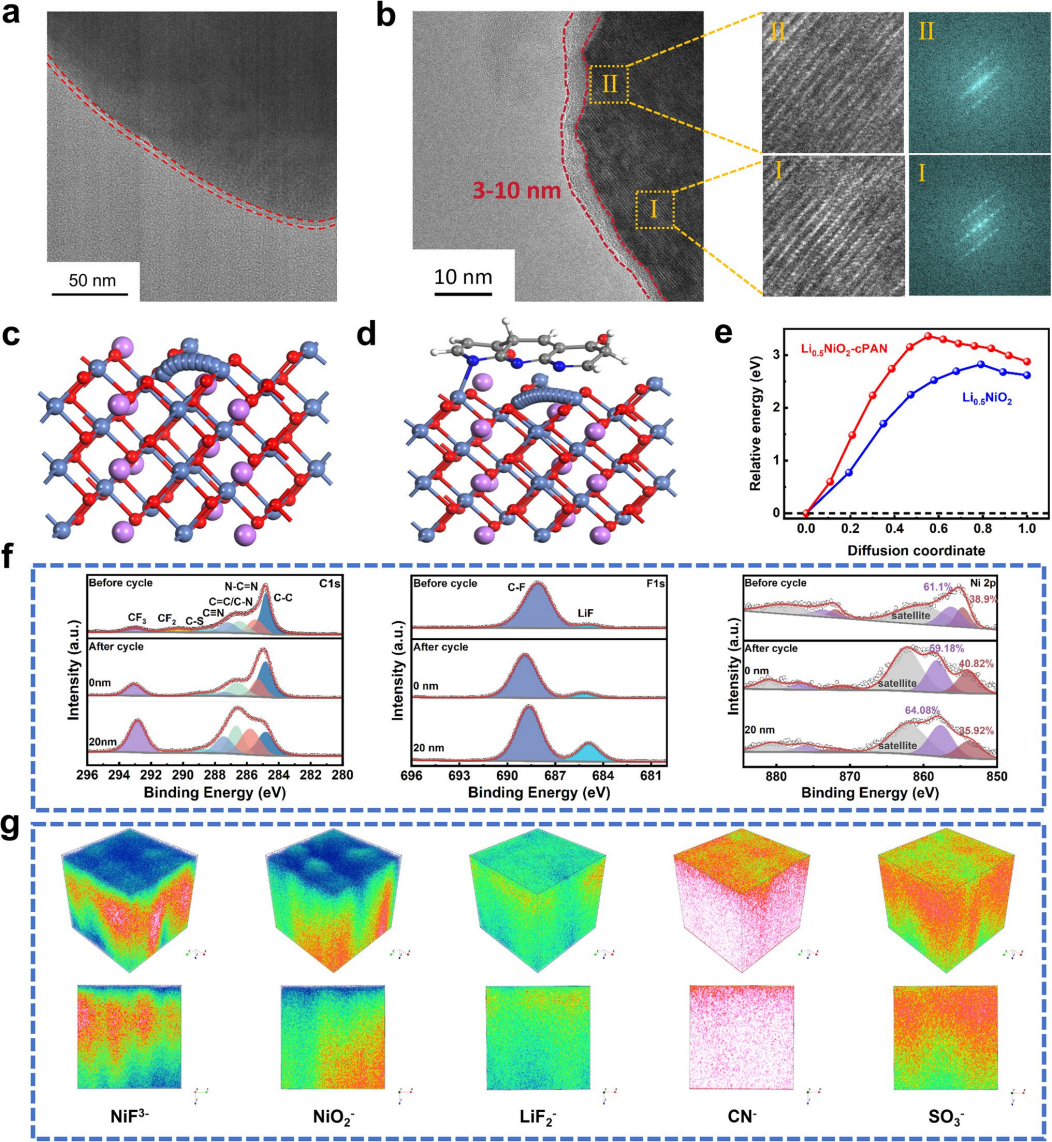
**a** TEM image and **b** cross-sectional SEM images of In-cPAN-260@NCM811 cathode after electrochemical cycling. **c-e** DFT simulation of transition metal (TM) ion migration process and calculation of energy barriers. **f** XPS spectra of In-cPAN-260@NCM811 cathodes before and after cycling. **g** TOF–SIMS analysis of cycled In-cPAN-260@NCM811 cathode

The crystal structure changes of In-cPAN-260@NCM811 particles after 50 cycles are shown in Fig. 4(a, b). There is still a uniform coating layer with a thickness of about 3–8 nm on the surface of particles after cycling, indicating that the coating layer will not fall off during the cycling. And In-cPAN-260@NCM811after 50 cycles still maintain layer structure. However, obvious rock salt phase can be detected on the surface of NCM811 in NCM811/SCPE/Li batteries after 50 cycles based on Fig. S14, indicating that coordinate bond between NCM811 and In-cPAN-260 coating layer is beneficial to maintain the stability of crystal structure. The Rietveld refinement patterns of In-cPAN-260@NCM811 before and after cycling are shown in Fig. S15. In-cPAN-260@NCM811 after cycling remains layer structure without the detection of impurity phase. It is worth noting that there is a peak locked at around 26°, which can be assigned to the binder PVDF. The lattice parameters of pristine and cycled In-cPAN-260@NCM811 are shown in Table S1, the similar cell parameters between pristine and cycled In-cPAN-260@NCM811 indicates that coating layer can maintain the stability of the crystalline structure effectively. Besides, the value of *I*_(003)_/*I*_(104)_ of In-cPAN-260@NCM811 basically remain unchanged after cycling, which can prove the protective effect of In-cPAN260 coating layer further [43, 44]. Generally, the value of *I*_(003)_/*I*_(104)_ is higher than 1.2, indicating a layered structure with a low degree of cation mixing [45]. In addition, (003)/(104) intensity ratios greater than 1.2 indicate a layered structure with a low degree of cation mixing and arranging. Therefore, In-cPAN-260@NCM811 presents low degree of cation mixing before and after cycling. To further elucidate the effect of coordination bond on the irreversible phase change, density functional theory (DFT) calculations were employed to simulate the migration process of transition metal (TM) ions and calculate energy barriers. At high voltage, massive Li^+^ is extracted to form large numbers of Li^+^ vacancies in the Li-layer. And Ni ions tend to migrate from TM-layer to the Li^+^ vacancies in the Li-layer at the highly delithiated state, resulting in the collapse of the crystalline structure and degradation of electrochemical performance. Therefore, the half delithiated state of LiNiO_2_ (Li_0.5_NiO_2_) acts as the structural model for Ni ions migration. In Fig. 4c–e, the energy barrier of Ni ions migration from TM-layer to Li-layer vacancies in cPAN@NCM811 (3.36 eV) is higher than that of NCM811 (2.83 eV), indicating that the coordination bond of Ni–N can hinder the migration of Ni. Thereby, the coordination bond interaction between NCM811 and cPAN can alleviate the collapse of the crystalline structure to enhance the electrochemical performance [46].

We further utilized XPS to investigate the compositional changes at the interface of pristine and cycled In-cPAN-260@NCM811. Figures 4f and S16 show the XPS spectra of NCM811 and In-cPAN-260@NCM811. The C-N (286.3 eV) in Fig. S16a is originated from the EMI^+^ cations in the composite cathode, CF_3_ (292.7 eV) and C-S (288.9 eV) are assigned to the FSI^−^ anions in the composite cathode, and CF_2_ (290.6 eV) belongs to the PVDF component in the composite cathode. After cycling, the C-N content of NCM811 exhibited a significant increase with 20 nm etching depth, suggesting that EMI^+^ underwent a decomposition reaction at the interface. And the increase of C-N content for In-cPAN-260@NCM811 is not significant as that of NCM811, indicating that cPAN-260 layer can effectively suppresses EMI^+^ decomposition. The C 1*s* spectrum of In-cPAN-260@NCM811 further demonstrates that the cPAN-260 layer does not fall off after cycling. In the F 1*s* spectrum of NCM811 and In-cPAN-260@NCM811, LiF (685 eV) is primarily derived from FSI^−^ decomposition. And the difference in LiF content between NCM811 and In-cPAN-260@NCM811 is not obvious, indicating that the coating layer has slight influence on the formation of LiF. Besides, the content of LiF increases significantly in the region close to the NCM811 surface, indicating that FSI^−^ is tent to decompose on the surface of NCM811 particles. Thus, cyclized polyacrylonitrile can form a stable interface layer with decomposition product of LiF due to the good oxidation resistance of In-cPAN-260 coating layer [47, 48]. At the same time, C = N bond and corresponding graphitization structure ensure rapid electron transfer in the interface layer before and after cycling. The peak positions of Ni^3+^ and Ni^2+^ are 856.2 and 854.7 eV [46], and the relative content of Ni^2+^ decreased from 41.4% to 38.9%, indicating that the close contact between the coating layer and NCM811 changed the surface chemical environment of the cathode material. And the content of Ni^2+^ in In-cPAN-260@NCM811 is lower than that of NCM811 after cycling, indicating that In-cPAN-260 layer can protect the crystal structure to alleviate the formation of rock salt phase. We also use TOF–SIMS to analyze the chemical composition of the CEI layer on the In-cPAN-260@NCM811 after cycling. Figure 4g shows the depth 3D and 2D distributions of several components of the CEI layer, including NiF_3_^−^, NiO_2_^−^, LiF_2_^−^, CN^−^, and SO_3_^−^ plasma fragments. Among them, there are less NiF_3_^−^, NiO_2_^−^, and MnF_3_^−^ on the surface, indicating that the relatively less dissolved transition metal ions [49–51]. The ion fragments of LiF_2_^−^ mainly originate from LiF, indicating that the CEI layer of the composite electrode contains LiF [52, 53] which comes from the decomposition of FSI^−^. The CN^−^ ion fragments derived from cyano and C = N bonds still have high strength and uniform distribution after cycling, which indicates that the coating formed by in situ polymerization of cyclization treatment has good electrochemical stability.

**Fig. 5:**
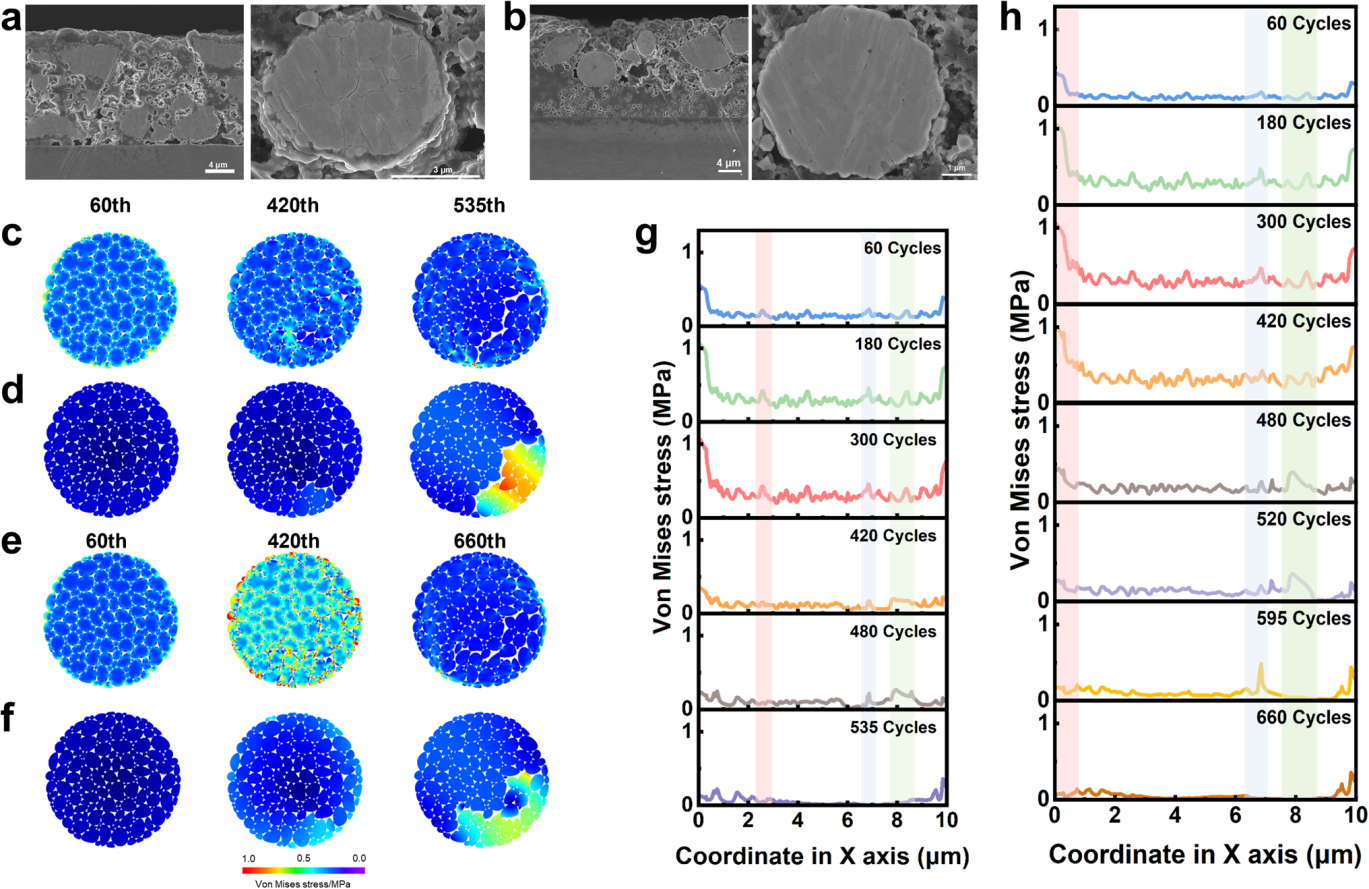
**a** Cross-sectional SEM images of NCM811 after electrochemical cycling.** b** Cross-sectional SEM images of In-cPAN-260@NCM811 after electrochemical cycling. **c** Visualization of the von Mises stress inside the NCM811 secondary of primary NCM811 at 60th, 420th, and 535th cycles. **d** Displacement of NCM811 after different cycles. **e** Visualization of the von Mises stress inside the NCM811 secondary of In-cPAN-260@NCM811 at 60th, 420th, and 660th cycles. **f** Displacement of In-cPAN-260@NCM811 after different cycles. **g** Distribution of von Mises stress inside NCM811 along the X-axis at Y = 5 μm after different cycles, the slide width is 1.0 μm. **h** Distribution of von Mises stress inside In-cPAN-260@NCM811 along the X-axis at Y = 5 μm after different cycles, the slide width is 1.0 μm

In addition, the cross-sections of the NCM811 and In-cPAN-260@NCM811 after 100 cycles were further observed, as shown in Fig. 5a, there are many cracks occur in NCM811 particles, which can be attributed to the uneven phase change. The phase change process leads to uneven distribution of stress within the grains, resulting in cracks within the grains, reducing the ionic and electronic conductivity of the material. However, the shape and structure of In-cPAN-260@NCM811 particles remain intact (Fig. 5b), indicating that the rigid structure cPAN coating layer can effectively inhibit the volume expansion of the cathode particles to ensures their structural integrity. In order to uncover the influence of cPAN coating layer microscopically and intuitively on the internal structure of NCM811 particles, COMSOL simulation was employed to reveal its stress variation process during cycling. The geometric model of NCM811 particles was abstracted through the superposition of different sizes of circular convex polygons. The cycling stress variation process of polycrystalline NCM811 was carried out in the same solid composite electrolyte environment, and the electrolyte was set as the isotropic continuum. Figure 5(c, d) shows the visualization of displacement and damage of the NCM811 cathode at different cycle times, of which the displacement of primary particles is strengthened. The stress of the NCM811 particles mainly concentrated on the interparticle boundary, which is gradually strengthened due to the volumetric strain of adjacent primary particles during cycling. Subsequently, NCM811 particles underwent intergranular collapse along the interparticle boundary, and the high stress region gradually shifted to the boundary of the cracked primary particles, further promoting particle fragmentation. The significant displacement would cause the loss of contact between adjacent primary particles within the NCM811 cathode particles. Therefore, cracks appeared at the interparticle boundary between adjacent primary particles and propagated along the interparticle boundary (Figs. S17(a, c). As shown in Figs. 5(e, f) and S17(b, d), In-cPAN-260@NCM811 occurs less displacement and cracks due to the presence of coating layer. Due to the repeated volume strain of primary particles during continuous cycling, cracks gradually developed into large fractures at the interparticle boundary. However, the formation of rigid structural network coating layer after PAN cyclized can effectively suppress the displacement of primary particles and generation of cracks in polycrystalline particles to maintain the integrity of polycrystalline particle structure, resulting in superior cycling stability. More detailly, the variations of von Mises stress inside NCM811 particles along X-axis were sliced at Y = 5 μm with the width of 1.0 μm (Fig. S18). As shown in Fig. 5(g, h) the stress distribution of NCM811 is gradually increasing and then disappear at 535th cycle, while the similar trend does not occur inside the In-cPAN-260@NCM811 at 520th and 595th cycles. The dissipation of stress can be attributed to the severe release of stress due to the disintegration of NCM811 secondary particles tolerated by the repetitive volumetric strain from the delithiation and lithiation process. By comparison, due to the strong mechanical limitation from rigid structural of cPAN, it is harder to displace locally for the primary particles and thus dissipate stress. On the other hand, the local displacement of coarse primary particles is less than the fine one due to the steric effect. Overall, the cPAN coating layer provides a rigid network structure and confined space that can effectively suppress significant cracks and displacements caused by uneven stress distribution in during cycling, maintain particle integrity, and improve the cycling stability of the battery.

### Thermal Safety Test

**Fig. 6:**
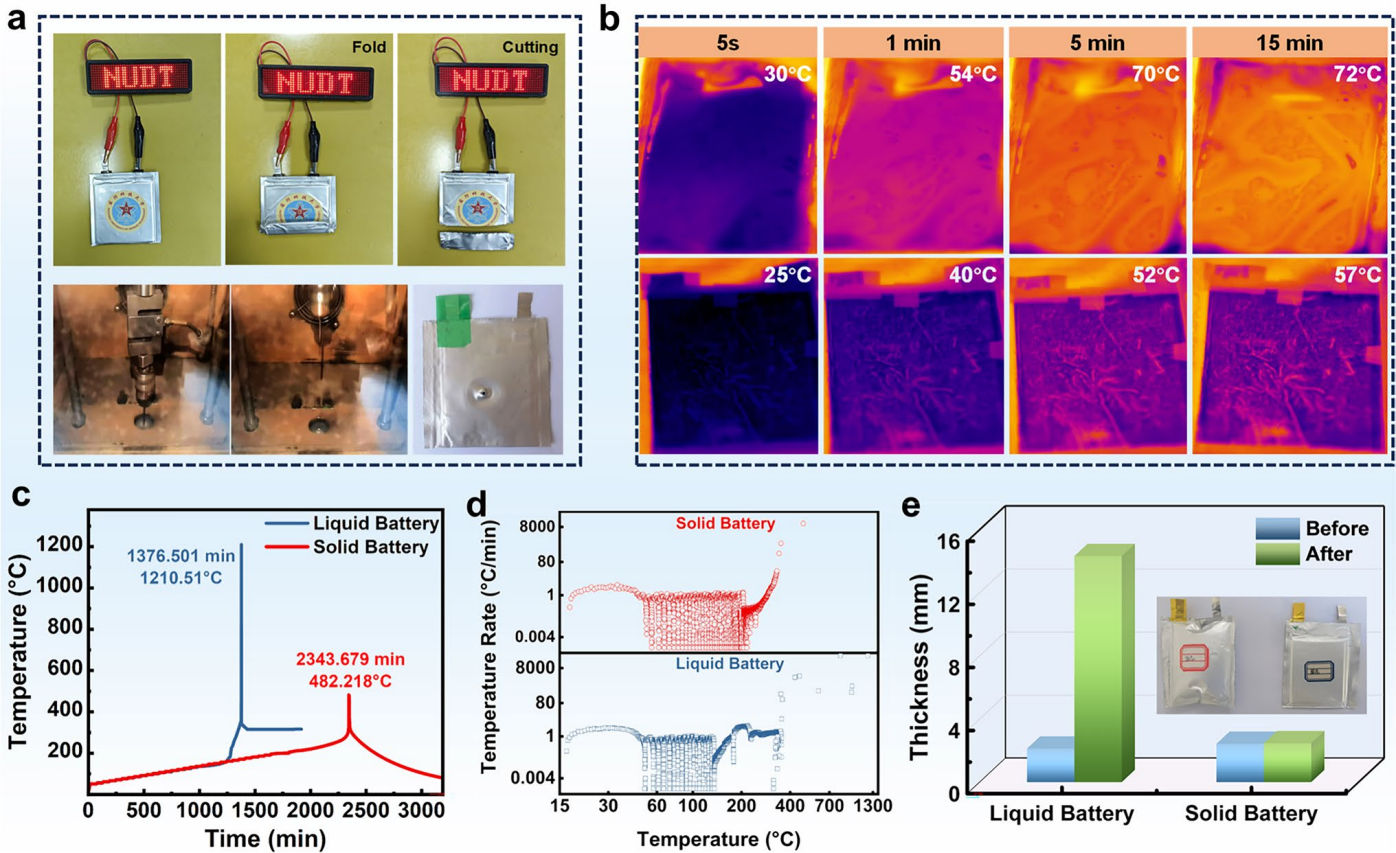
**a** Folding and cutting display of soft-pack battery and needle puncture experiment. **b** Thermal infrared imaging test of liquid- and solid-state soft-pack batteries. **c** ARC test results of liquid batteries and solid batteries. **d** Temperature rate of liquid battery and solid battery. **e** Thermal expansion test of liquid battery and solid battery

Ultimately, the laminated pouch cell was assembled to verify the application feasibility and safety evaluation of the In-cPAN-260@NCM811 solid-state battery under harsh conditions. As shown in Fig. 6a, the fully charged pouch cell could light up the LED lamp in “NUDT” pattern with strong luminescence even after folding or cutting. Additionally, when the steel needle stabbed into the fully charged solid-state battery, the battery did not leakage, fire and explode, demonstrating that the good safety performance of solid-state battery. Additionally, the temperature change of liquid battery and solid-state battery with time at a certain heating temperature is measured by infrared thermal imager. As shown in Fig. 6b, the temperature of liquid battery rises from room temperature to about 70 °C in only 5 min and solid-state battery presents a lower temperature, indicating the higher risk of thermal runaway of liquid battery due to the rapid heat transfer. Therefore, the solid-state battery has higher safety and stability due to their obstructed heat transfer and a relative decrease in battery system temperature.

In addition, the thermal stability of solid-state battery and conventional organic electrolyte battery was studied by accelerated calorimeter (ARC) in adiabatic environment. The accelerated calorimeter (ARC) test further proves the thermal safety of solid-state batteries. As shown in Fig. 6c, d, the liquid battery has a self-exothermic reaction at about 160.9 °C, and the maximum temperature is 1210.51 °C at the time of thermal runaway (1376.501 min). However, the temperature of the solid-state battery starts self-heating is 255.6 °C, and there is also a delay in reaching the highest temperature (2343.679 min and 482.218 °C), reflecting the good thermal safety stability. More notably, as shown in Fig. 6e, the ratio of volume expansion of the solid-state pouch cells can be ignored after the thermal expansion test (store at 90 °C for 3 h), However, the volume of the liquid battery expanded rapidly and the thickness increased from 2.126 to 14.33 mm after the thermal expansion test, indicating that solid-state battery has good high-temperature stability to avoid the safety issues. Accordingly, the above test results show that replacing liquid electrolyte with solid electrolyte can effectively reduce heat release, delay the occurrence of exothermic reaction, and improve the thermal stability and safety of the battery.

## Conclusions

In summary, a continuous and uniform ion electron mixing conductive modified layer is introduced on the surface of NCM811 by in situ polymerization of PAN and cyclization heat treatment. The DFT results show that the coordination between the polar group cyano in PAN and transition metal ions can promote the close contact between the coating and particles, enhance the stability of crystal structure, and reduce the interfacial side reactions. This mixed conductive interface layer can construct fast transfer channel of the electron and ion in the composite solid-state cathode, accelerating the interface charge transfer speed and significantly improving the rate performance. In addition, the interface layer effectively stabilizes the cathode–electrolyte interface through the oxidation resistance of cyclized polyacrylonitrile and LiF produced by the decomposition of LiFSI during the cycling. At the cutoff voltage of 4.3 V, In-cPAN-260@NCM811 achieves excellent cycling stability with a capacity retention rate of 86.8% after 300 cycles at 60 °C. Especially, In-cPAN-260@NCM811 can operate stably at 100 °C with a capacity retention rate of 75% after 100 cycles. It provides theoretical support for the development of solid-state lithium battery system with safe, reliable, and good comprehensive performance even at high-temperature extreme environment.

## Supplementary Information

Below is the link to the electronic supplementary material.

Supplementary file1 (DOCX 6383 KB)
