# Supplementary material for: In Situ Partial-Cyclized Polymerized Acrylonitrile-Coated NCM811 Cathode for High-Temperature ≥ 100 °C Stable Solid-State Lithium Metal Batteries
Source: Nanomicro Lett. 2025 Mar 19;17:195. doi: 10.1007/s40820-025-01683-7 (PMC11923343; doi:10.1007/s40820-025-01683-7)
Supplement: Supplementary file 1 — Supplementary file1 (DOCX 6383 KB) [file 40820_2025_1683_MOESM1_ESM.docx]

**Supporting information**

**In-situ Partial-cyclized Polymerized Acrylonitrile-coated NCM811 Cathode for High-temperature ≥100 ℃ Stable Solid-state Lithium Metal Batteries**

*Jiayi Zheng^1,+^,Haolong Jiang^1,+^, Xieyu Xu^2,+^,Jie Zhao^1^, Xia Ma^1^, Weiwei Sun^1^, Shuangke Liu^1^, Yufang Chen^1^, ShiZhao Xiong^3^, Hui Wang^1^,* *Kai Xie^1^, Yu Han^1,^*, Chunman Zheng^1,^*, Maoyi Yi^1,^*, Qingpeng Guo^1,^**

1 College of Aerospace Science and Engineering, National University of Defense Technology, Changsha, Hunan 410073, China

2 State Key Laboratory for Mechanical Behavior of Materials, Xi'an Jiaotong University, Xi'an, Shanxi 710049, China

3 Faculty of Materials Science and Engineering, Kunming University of Science and Technology, Kunming, 650093 China

+ These authors contributed equally.

*Corresponding author. E-mail:

yumihan1981@sina.com (Y. Han)

zhengchunman@nudt.edu.cn (C. Zheng)

yimaoyi_1993@163.com (M. Yi)

qingpeng.guo@nudt.edu.cn (Q. Guo)

**Supporting Tables and Figures**

**Table S1** Rietveld refinement parameters of XRD patterns for In-cPAN-260@NCM811 before and after cycling

| Sample | a (Å) | c (Å) | Volume (Å) | I_(003)_/I_(104)_ |
| --- | --- | --- | --- | --- |
| Before cycling | 2.8709 | 14.1958 | 101.33 | 2.29 |
| After cycling | 2.8737 | 14.2385 | 101.83 | 2.25 |


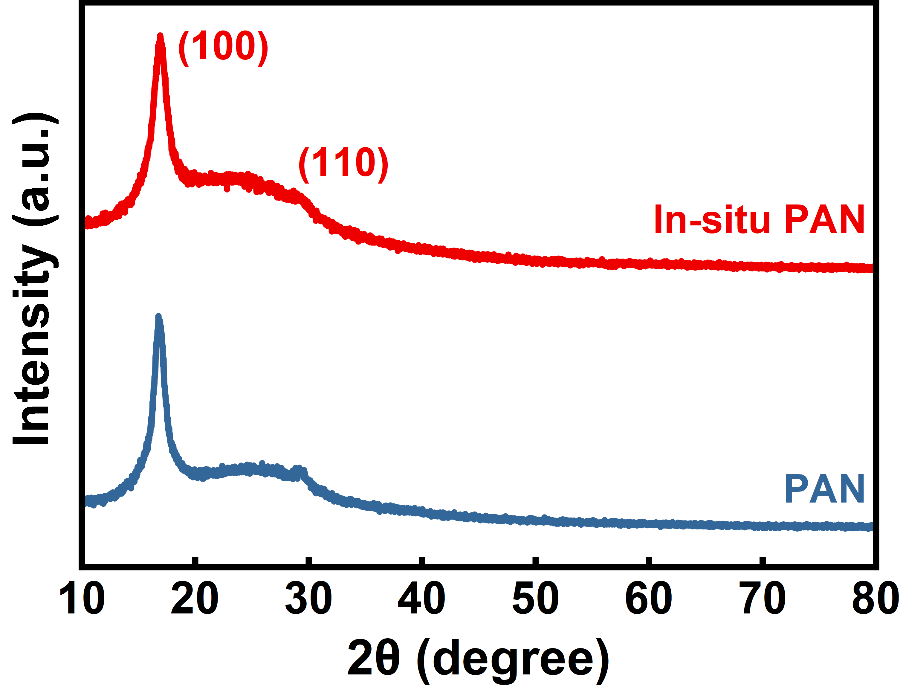


**Fig. S1** XRD pattern of PAN and In-situ PAN.


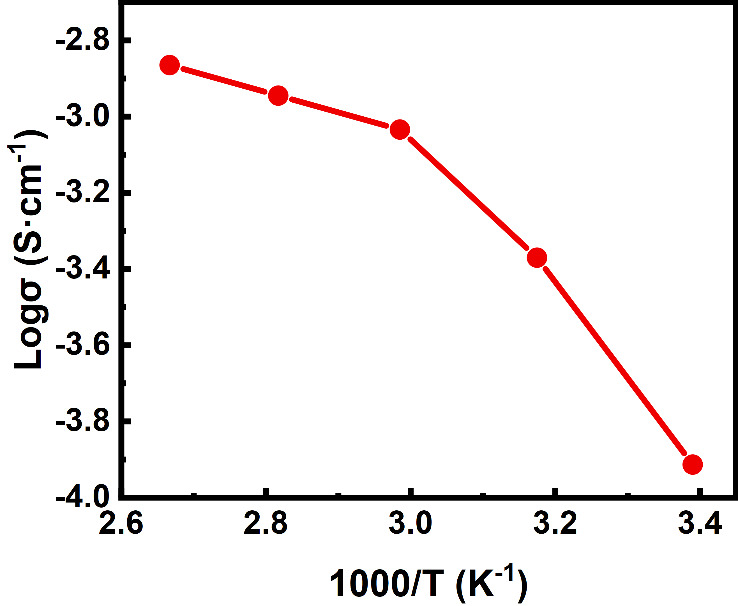


**Fig. S2** Arrhenius plot of ionic conductivity versus temperature for PAN electrolytes.


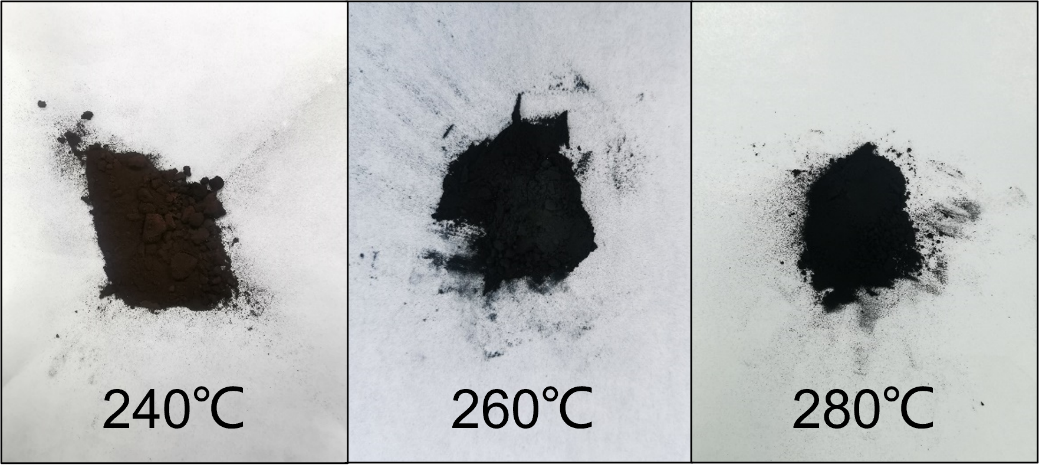


**Fig. S3** Optical photographs of cPAN formed at different sintering temperatures.


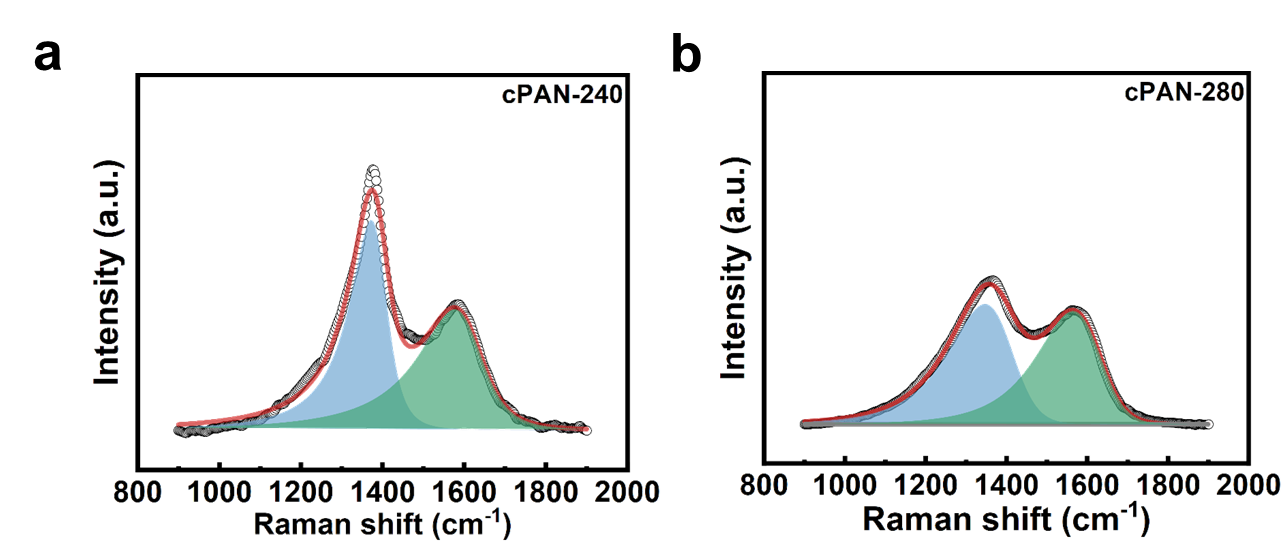


**Fig. S4** Raman spectra of cPAN-240 and cPAN-280.


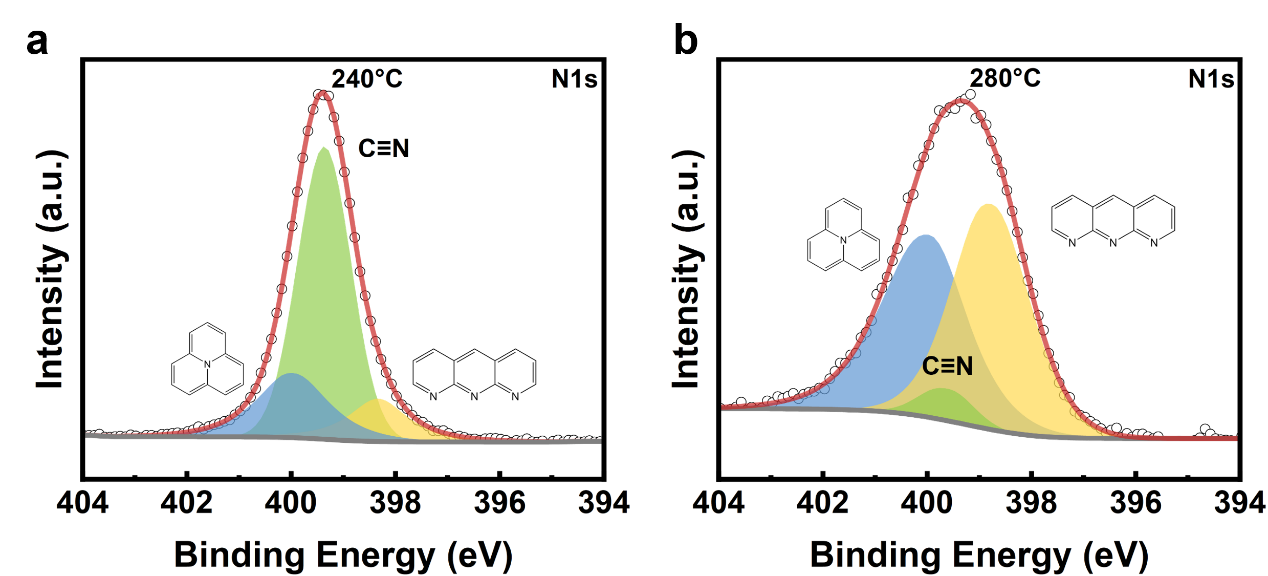


**Fig. S5** XPS spectra of cPAN-240 and cPAN-280.


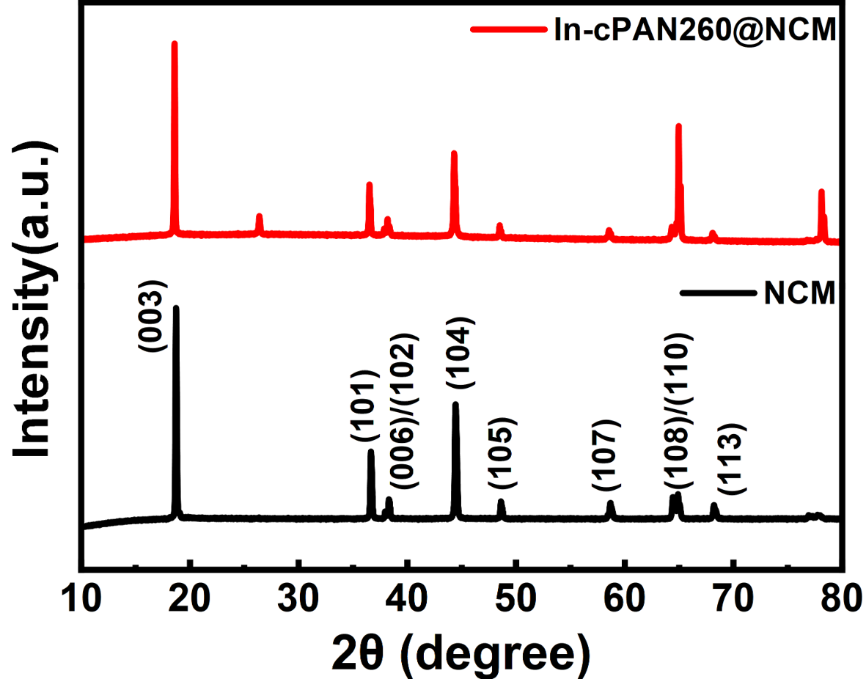


**Fig. S6** XRD pattern of NCM811 and In-cPAN@NCM811.


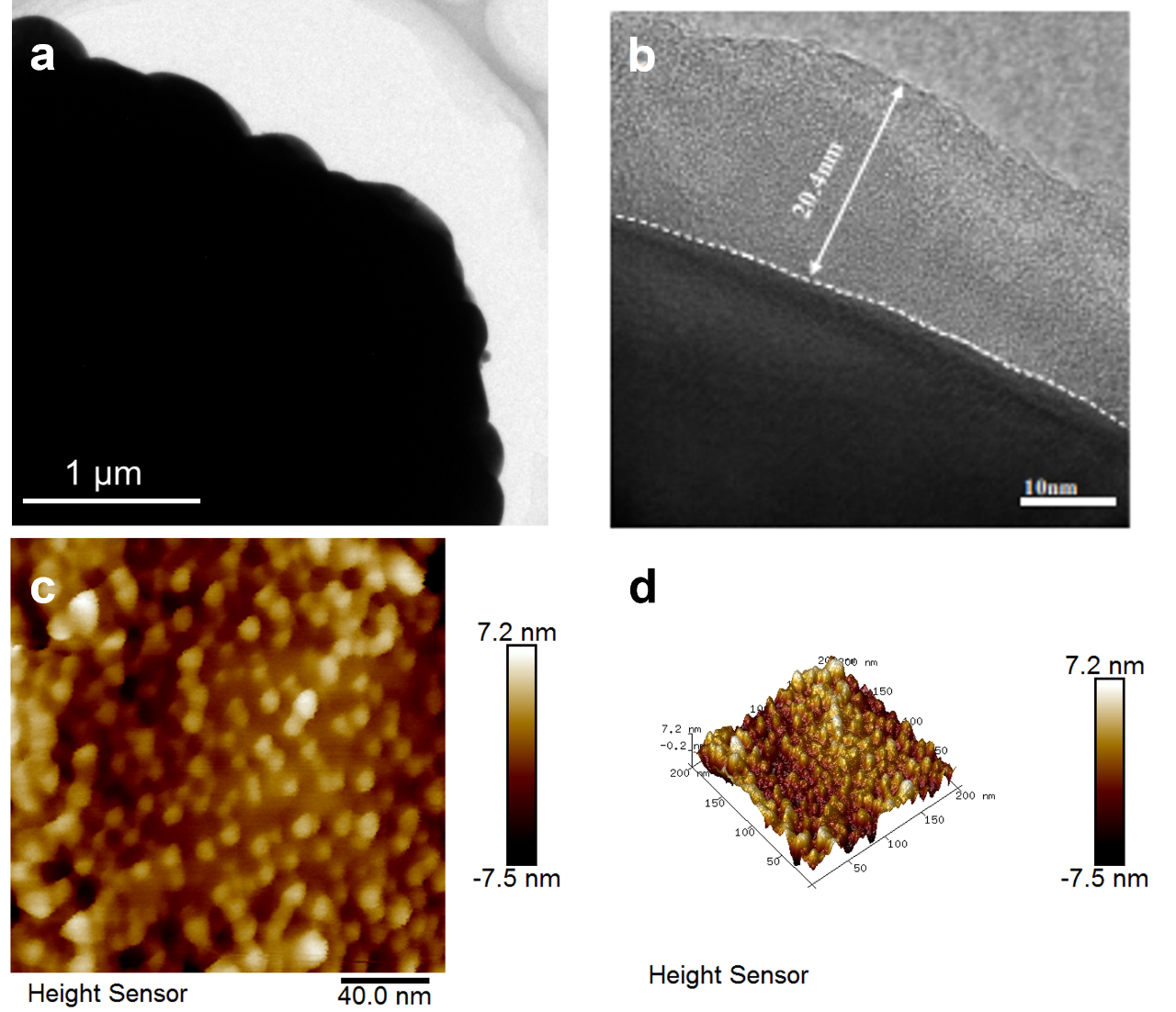


**Fig. S7** TEM images of (a) pristine NCM811 and (b) PAN@NCM811[1], (c)-(d) AFM images of pristine NCM811. (e)-(f) In-cPAN-260@NCM811.


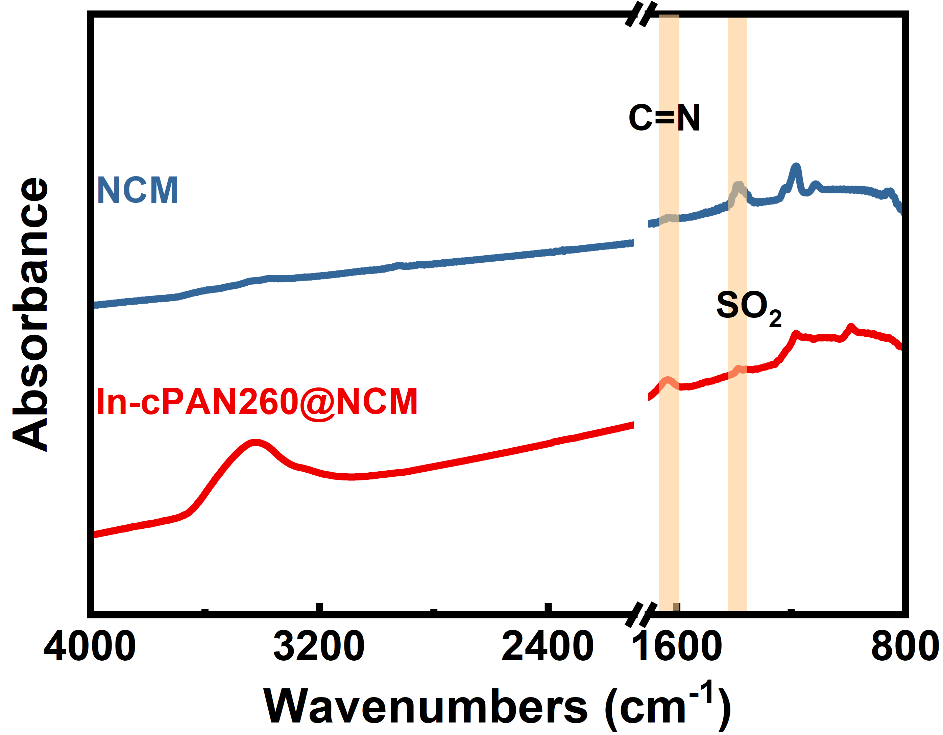


**Fig. S8** FTIR pattern of NCM811 and In-cPAN-260@NCM.


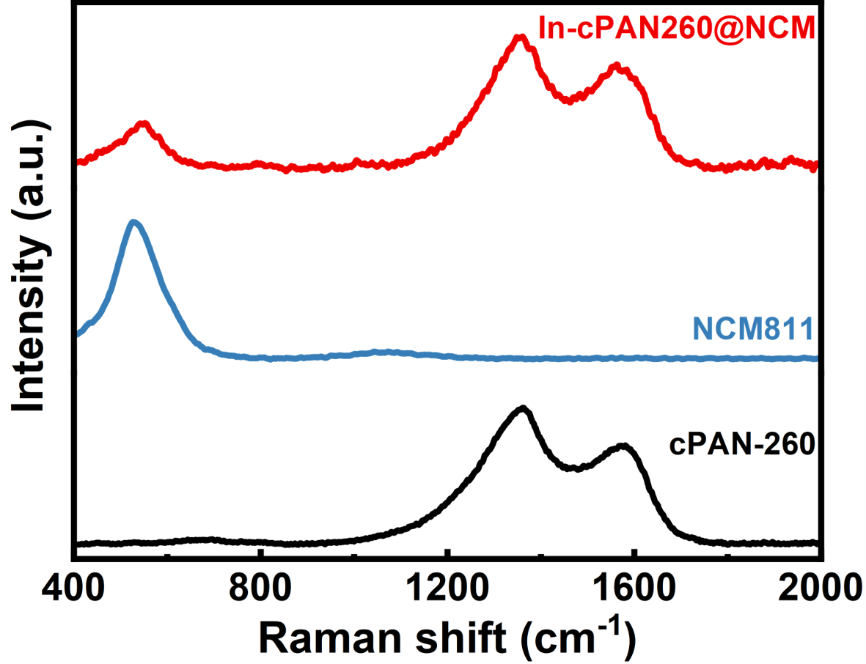


**Fig. S9** Raman spectra of cPAN-260, NCM811 and In-cPAN-260@NCM811.


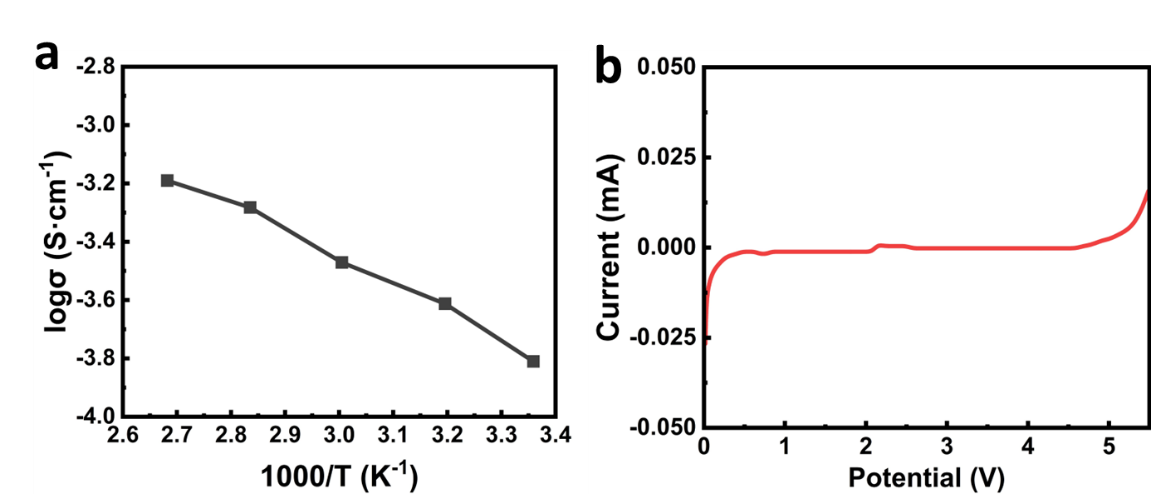


**Fig. S10** **a** Arrhenius plot of ionic conductivity versus temperature for PVDF-HFP-based electrolyte. **b** LSV curves of PVDF-HFP-based electrolyte.





**Fig. S11** Long cycling of PVDF-HFP-based electrolyte in symmetric cells at 0.05 mA·cm^−2^ and 0.05 mAh·cm^−2^.


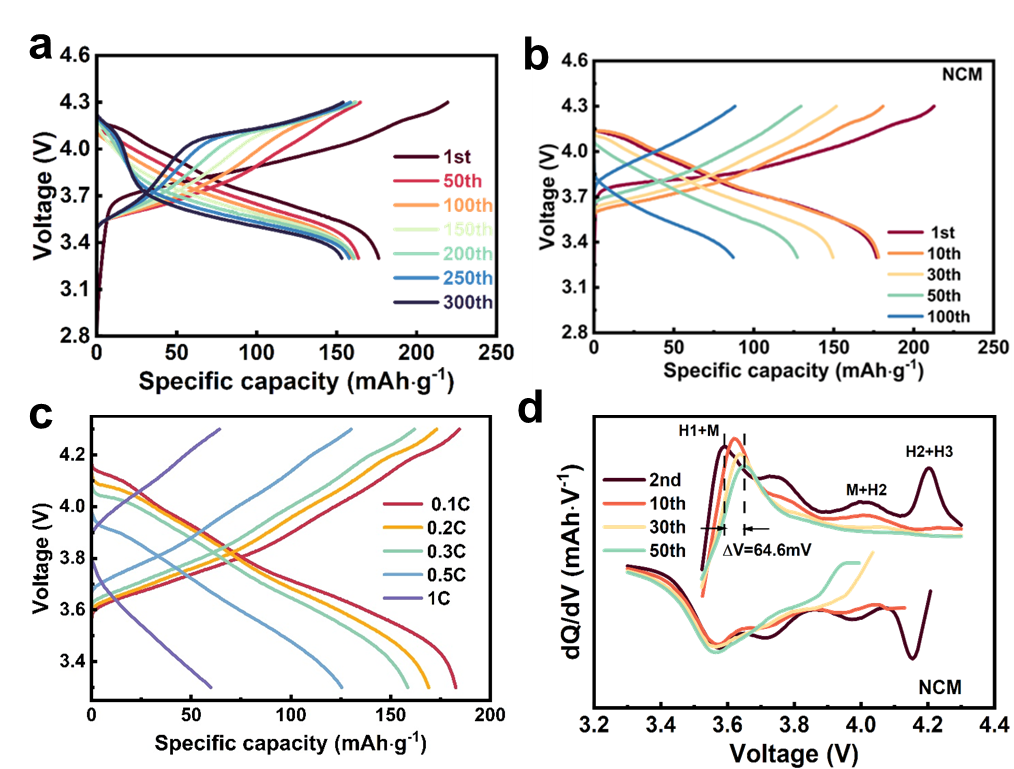


**Fig. S12** **a** Charge and discharge curves of In-cPAN-260 @NCM811. **b** Charge and discharge curves of NCM811. **c** Charge and discharge curves of NCM811 at different current density. **d** The dQ/dV curves of NCM811.


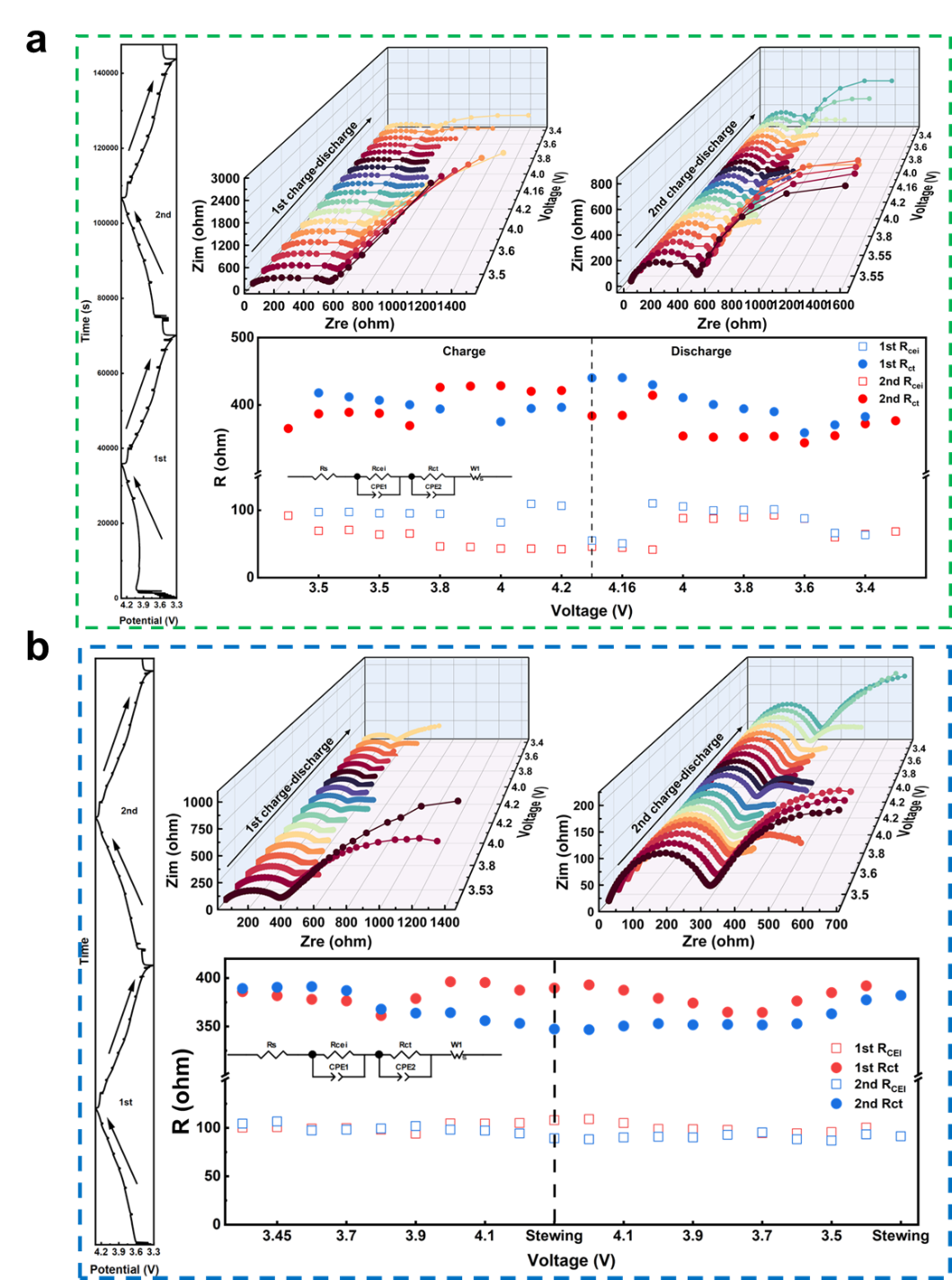


**Fig. S13** In situ impedance spectra of **a** NCM811 and **b** In-cPAN-260@NCM811.


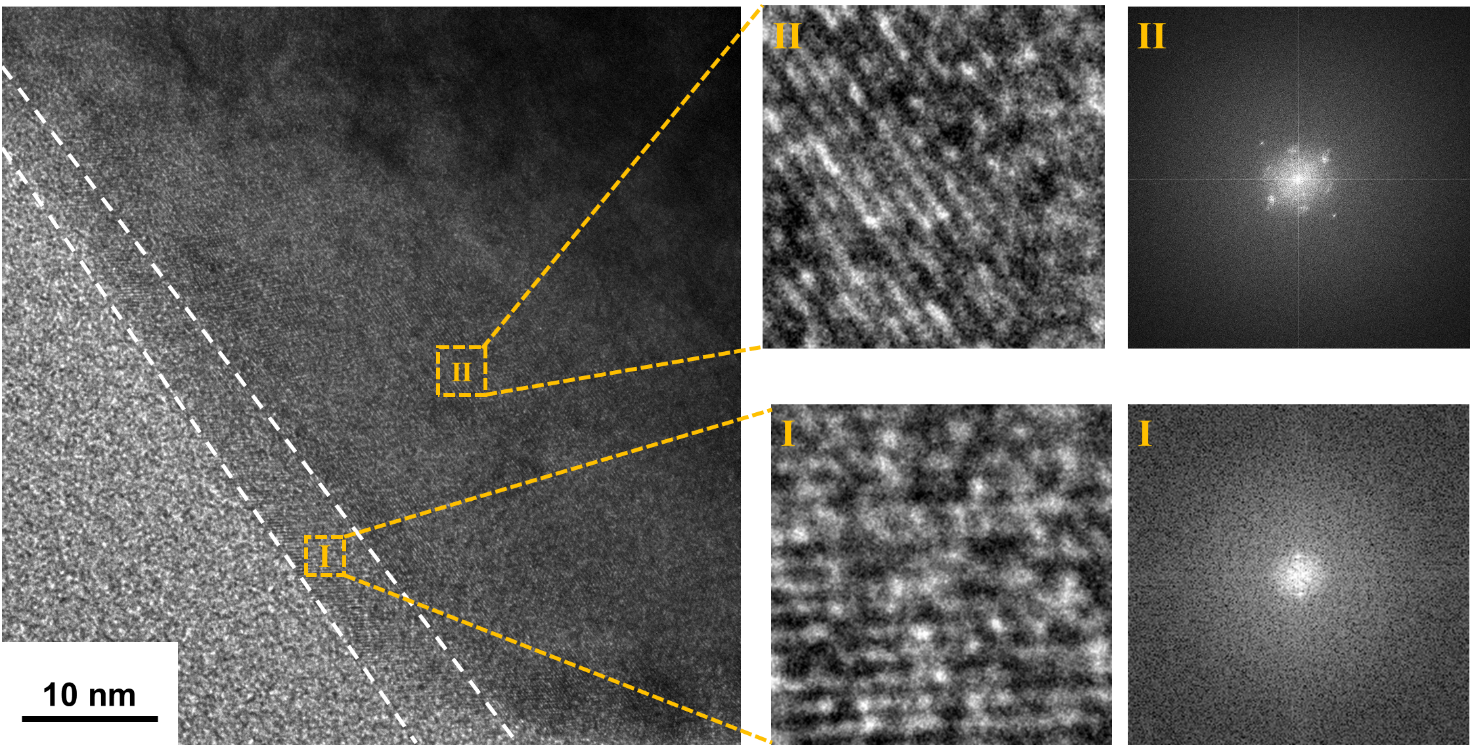


**Fig. S14** TEM images of NCM811 cathode in NCM811/SCPE/Li batteries after 50 cycles.


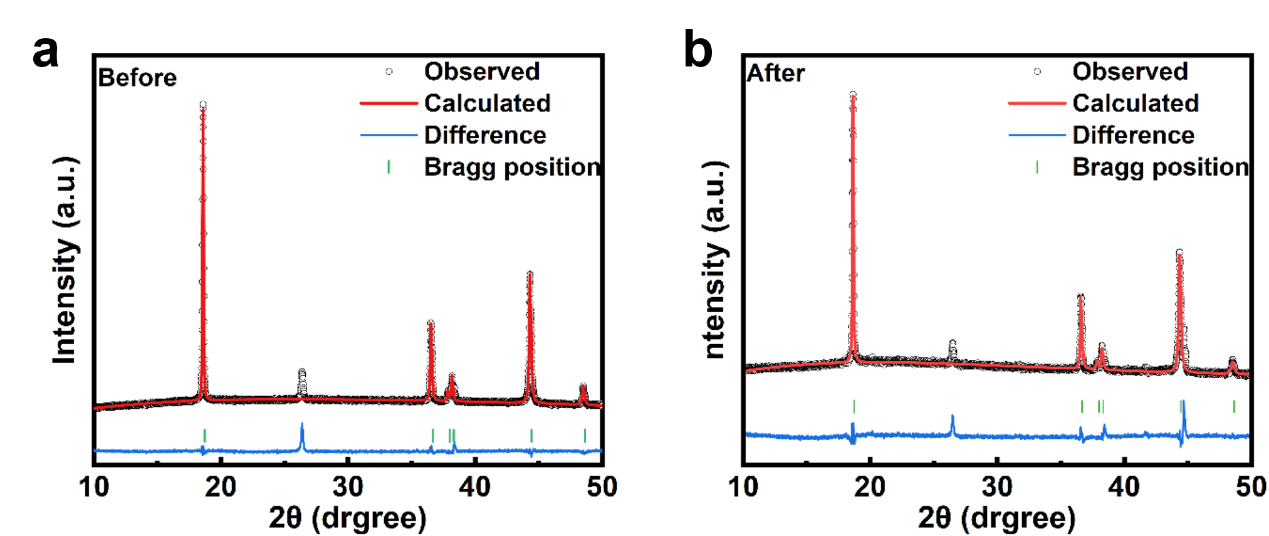


**Fig. S15** XRD Rietveld refinement patterns of In-cPAN-260@NCM811 before and after cycling.


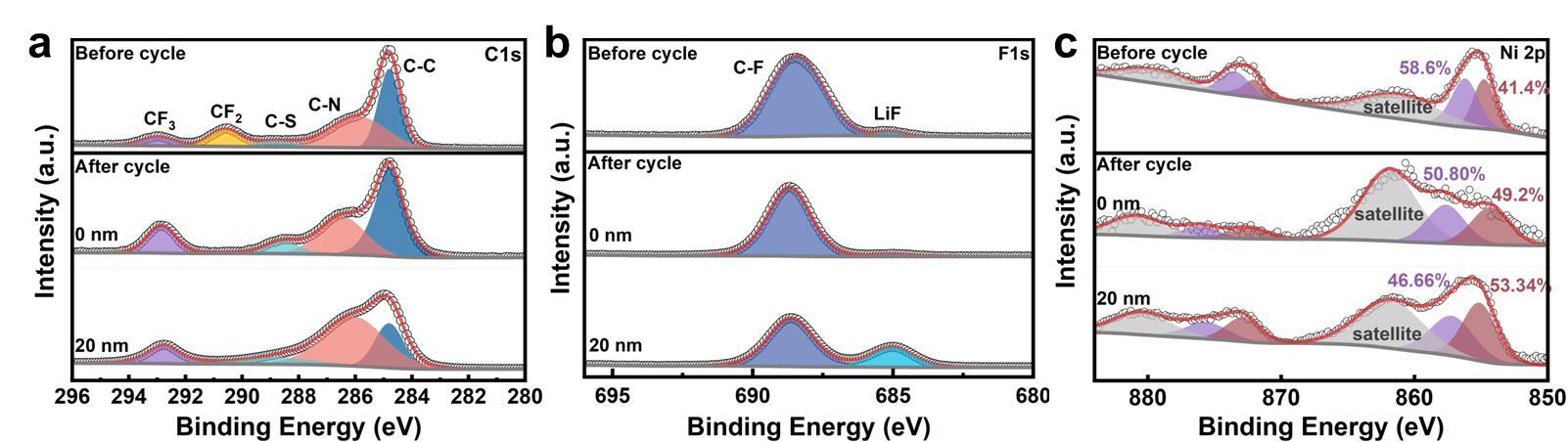


**Fig. S16** XPS spectra of NCM811 cathode before and after cycling: (a) C1s; (b) F1s; (c) Ni2p.


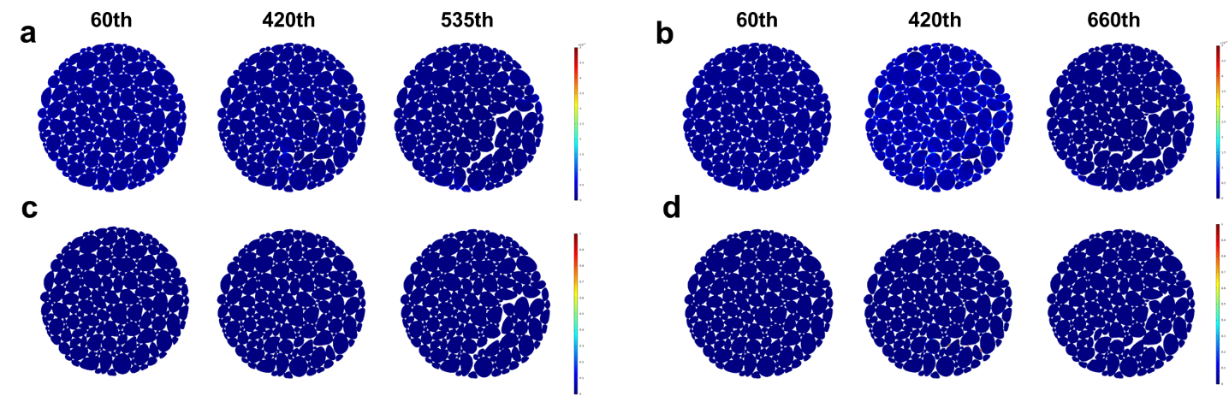


**Fig. S17** Visualization of cracks inside the NCM811 secondary of **a** NCM811 and **b** In-cPAN-260@NCM811 at different cycles.Visualization of damage inside the **c** NCM811 and **d** In-cPAN-260@NCM811 at different cycles.


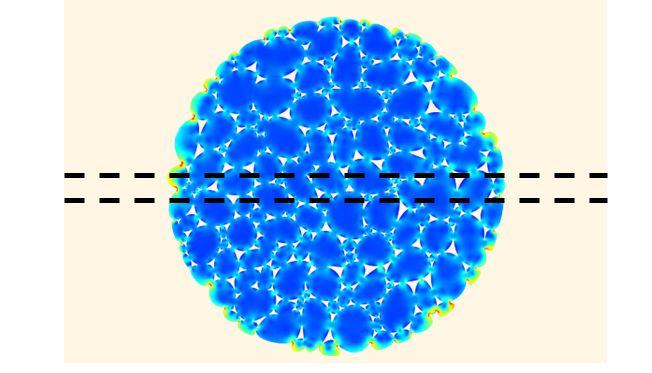


**Fig. S18** Distribution of von Mises stress along the X-axis at Y=5 μm after different cycles, the slide width is 1.0 μm

**Supporting References**

[1] H. Jiang; X. Xu; Q. Guo; H. Wang; J. Zheng et al., Electro-chemo-mechanical design of polymer matrix in composited LiNi_0.8_Co_0.1_Mn_0.1_O_2_ cathode endows solid-state batteries with superior performance. J. Energy Chem. **78**, 277 (2023). <http://doi.org./10.1016/j.jechem.2022.11.059>
